# Site- and Compartment-Associated Differentiation of Root Microbiomes in *Rhododendron yunnanense*

**DOI:** 10.3390/microorganisms14102264

**Published:** 2026-10-05

**Authors:** Jian Dong, Zhaosheng Gao, Liu Yang, Yawen Wu, Tian Bai, Yan Dong, Jingli Zhang

**Affiliations:** 1 College of Landscape and Horticulture, Yunnan Agricultural University, Kunming 650201, China; 2 China Flower Association, Beijing 100020, China

**Keywords:** *Rhododendron yunnanense*, root microbiome, rhizosphere, endosphere, site heterogeneity

## Abstract

Root-associated microbiomes vary across environmental settings and root compartments, but these patterns remain poorly understood in wild woody plants. Here, we compared bacterial and fungal communities in the rhizosphere and root endosphere of *Rhododendron yunnanense* across four montane sampling sites in Yunnan, China, using 16S rRNA gene and internal transcribed spacer (ITS) amplicon sequencing. α-diversity differences were mainly reflected in amplicon sequence variant (ASV) richness, whereas Shannon and Simpson diversity varied less among site–compartment combinations. Bray–Curtis community composition was significantly associated with sampling site, root compartment, and their interaction for both bacteria and fungi. Stable and core ASV analyses further showed limited overlap between the rhizosphere and endosphere. Under the predefined core criteria, only three bacterial ASVs were shared between compartments, while no fungal ASV qualified as a shared core member. The extent of stable-ASV overlap varied among sampling sites and differed between bacterial and fungal communities. Indicator-value analysis, paired abundance testing, and Random Forest classification converged on a smaller set of compartment-associated genera. Thirteen taxonomically interpretable genera were retained for main-text interpretation, including five bacterial genera associated with the endosphere and eight fungal genera, of which *Phialocephala* was associated with the rhizosphere. Together, these results show that sampling site and root compartment are both associated with marked differentiation of the *R. yunnanense* root microbiome. The low overlap of stable and core taxa further indicates substantial microbial turnover between the rhizosphere and endosphere, providing a basis for future functional validation of compartment-associated taxa.

## 1. Introduction

Plants and their associated microorganisms form closely linked biological systems that play important roles in plant growth and responses to environmental conditions. Plants can influence the structure and composition of microbial communities in the rhizosphere and internal tissues, while associated microorganisms may contribute to host performance through nutrient acquisition, pathogen suppression, defence responses, and secondary metabolite-related processes [1,2,3,4]. Root-associated microbiomes are particularly important because roots represent a major interface between plants and their surrounding soil environment. However, documenting the ecological importance of plant-associated microbiomes is only a first step. A more fundamental question is how these communities are differentiated and assembled across root compartments under contrasting environmental conditions.

Root exudates and root-derived organic inputs provide carbon and nutrient resources that contribute to the formation of distinct microbial sampling sites around roots [5,6]. Rhizosphere microbial communities are jointly associated with soil physicochemical conditions and plant root activity. Among environmental factors, soil pH has frequently been identified as an important correlate of bacterial and fungal community structure, while soil nutrients such as nitrogen and available phosphorus may also contribute to variation in microbial diversity and composition [7,8,9,10]. Elevation-associated environmental variation has likewise been linked to changes in fungal and bacterial communities in mountain ecosystems [11]. At the same time, plant roots modify the immediate microbial sampling site through exudation and other rhizosphere processes [12]. Together, these findings suggest that root-associated microbial communities emerge from the combined influence of environmental context and host compartment, although their relative patterns may vary among microbial groups, host species, and sampling sites.

Mountain ecosystems provide useful natural systems for examining microbial community variation because elevation is often accompanied by substantial changes in climate, vegetation, and soil conditions. Previous studies have reported contrasting elevational patterns in fungal and bacterial communities, including monotonic, unimodal, and non-significant responses, indicating considerable context dependency among mountain systems [13,14,15,16,17,18]. In *Rhododendron* ecosystems, bacterial and fungal diversity in the rhizosphere has also been shown to vary along elevational gradients [19]. However, most previous studies have focused on bulk soil or rhizosphere communities, whereas coordinated comparisons of rhizosphere and endosphere microbiomes remain relatively limited, particularly in woody montane plants.

Studies of root microbiomes have shown that microbial communities associated with the rhizosphere and roots can differ substantially in composition [20,21]. However, in *Rhododendron* growing across mountain environments, relatively little is known about whether these compartment-associated differences are consistent among geographically distinct sampling sites. Another important question is how microbial community membership differs between the rhizosphere and root compartment, including which taxa are shared, which are compartment specific, and which differ in relative abundance. Comparisons of shared, stable, and core taxa, together with compartment-associated indicator taxa, provide a useful way to describe these patterns without assuming direct microbial movement between compartments.

*Rhododendron yunnanense* is an evergreen shrub or small tree of the Ericaceae that is widely distributed in montane regions of southwestern China [22,23]. It commonly occurs on mountain slopes at elevations of approximately 2200–3600 m and is distributed in Yunnan, Sichuan, Guizhou, Shaanxi, Tibet, and parts of northeastern Myanmar [23]. Its broad distribution across mountain sampling sites makes it a suitable species for examining variation in root-associated microbial communities under different environmental conditions. Previous studies of *Rhododendron* microbiomes have mainly examined soil or rhizosphere-associated communities along environmental gradients, whereas paired comparisons of bacterial and fungal communities between the rhizosphere and root compartments across natural montane sampling sites remain limited. In addition, relatively little is known about the consistency of shared, stable, and core microbial taxa across these compartments in wild *Rhododendron*.

To address these questions, we compared bacterial and fungal communities in the rhizosphere and endosphere of wild *R. yunnanense* from four sampling sites in Yunnan Province. We first examined differences in microbial diversity, community structure, and taxonomic composition among sampling sites and between root compartments. We then compared shared, stable, and core ASVs to describe differences in community membership between the rhizosphere and root compartment and to assess patterns of compartment-associated differentiation. Finally, indicator-value analysis, nonparametric abundance comparisons, and Random Forest classification were used together to identify bacterial and fungal taxa that were consistently associated with either the rhizosphere or the endosphere. Through these analyses, we aimed to better understand how sampling site and root compartment are associated with the differentiation of root-associated microbial communities in *R. yunnanense*.

## 2. Materials and Methods

### 2.1. Plant Material

Four sampling sites of *Rhododendron yunnanense* were selected in Yunnan Province, China: Junzishan Mountain, Shizong County, Qujing City (J; 104°9′49.3″ E, 24°38′22.9″ N; 2252 m a.s.l.); Zhujiangyuan, Zhanyi District, Qujing City (Z; 103°56′26.5″ E, 25°53′39.4″ N; 2305 m a.s.l.); Ailao Mountain Scenic Area, Jingdong County, Pu’er City (A; 101°1′15.6″ E, 24°31′15.6″ N; 2483 m a.s.l.); and Huadianba, Cangshan Mountain, Dali City (C; 100°2′17.2″ E, 25°53′12.1″ N; 2601 m a.s.l.) (Table S1). The sites covered an elevation range of 2252–2601 m and represented geographically distinct montane sampling sites with contrasting local environmental conditions (Figure S1A).

The four sites also differed in surrounding vegetation and local environmental conditions. At Junzishan, *R. yunnanense* occurred together with species such as *Rhododendron delavayi* and *Quercus aliena* var. *acutiserrata*, whereas *Rhododendron irroratum* and *Rhododendron decorum* were common associated species at Zhujiangyuan. Zhujiangyuan and Cangshan were generally more humid, while Junzishan and Ailao Mountain were relatively drier. These differences provided the environmental background for comparing root-associated microbial communities among the four sites.

Sampling was conducted from 10 to 20 November 2023. At Junzishan, four individual *R. yunnanense* plants were sampled, whereas three individual plants were sampled at each of the other three sites. Root samples consisted of visually selected fine roots associated with each sampled plant. Exact root diameter and root order were not recorded during field sampling. Corresponding rhizosphere soil was collected from the same sampled plants. Rhizosphere samples were collected following the procedure described by Freschet and Roumet [24]. Root samples intended for characterization of the root interior microbiome were rinsed with distilled water before DNA extraction. No chemical surface-sterilization treatment or independent verification of surface sterility was performed. Therefore, the term “endosphere” is used cautiously in this study to refer to the sampled root interior compartment and does not imply complete exclusion of surface-associated microorganisms. Rhizosphere and endosphere samples sharing the same site and replicate number were treated as matched pairs At Junzishan, four matched rhizosphere–endosphere pairs were available, whereas three matched pairs were available at each of the other three sampling sites. To maintain equal biological replication among sampling sites in the final comparative analyses, three matched pairs from Junzishan were retained and the fourth pair was excluded from the final statistical analyses. Thus, three matched rhizosphere–endosphere pairs from each sampling site were included in the final analyses, resulting in 24 samples for bacterial analysis and 24 samples for fungal analysis. Samples were transported on ice, flash-frozen in liquid nitrogen, and stored at −80 °C until DNA extraction.

### 2.2. Soil Physicochemical Properties

Rhizosphere soil used for physicochemical characterization was collected from the 10–20 cm soil layer associated with the sampled *R. yunnanense* plants. Three independent rhizosphere soil samples were analyzed at each sampling site. Site-level values reported in Table S2 and Figure S2 represent the mean ± SD of these three replicate measurements. Soil pH was measured in a 1:2.5 soil-to-water suspension using a PHS-3E pH meter (INESA, Shanghai, China). Total nitrogen (TN) was determined by the Kjeldahl method, total phosphorus (TP) by alkaline digestion followed by molybdenum–antimony colorimetry, and total potassium (TK) by NaOH fusion followed by flame photometry. Available potassium (AK) was extracted with neutral ammonium acetate and measured by flame photometry according to NY/T 889-2004 [25]. Hydrolysable nitrogen (HN) was determined using the alkaline hydrolysis diffusion method (LY/T 1228-2015) [26], and available phosphorus (AP) was extracted using the hydrochloric acid–sulfuric acid method (LY/T 1232-2015) [27]. Soil organic matter (SOM) was measured using potassium dichromate–sulfuric acid oxidation titration according to NY/T 1121.6-2006 [28]. The procedures for the main soil properties followed Bao (2000) [29].

### 2.3. DNA Extraction, PCR Amplification and Sequencing

Total genomic DNA was extracted from rhizosphere soil and root samples using the OMEGA Soil DNA Kit (D5635-02; Omega Bio-tek, Inc., Norcross, GA, USA) according to the manufacturer’s instructions. DNA integrity was checked by 0.8% agarose gel electrophoresis, and DNA concentration and purity were measured using a NanoDrop spectrophotometer (Thermo Fisher Scientific, Waltham, MA, USA).

The V3–V4 region of the bacterial 16S rRNA gene was amplified using primers 338F (5′-ACTCCTACGGGAGGCAGCA-3′) and 806R (5′-GGACTACHVGGGTWTCTAAT-3′). The fungal ITS1 region was amplified using primers ITS1 (5′-CTTGGTCATTTAGAGGAAGTAA-3′) and ITS2 (5′-GCTGCGTTCTTCATCGATGC-3′). PCR amplification was performed in 20 μL reactions containing 5–50 ng of DNA template, 0.3 μL of each primer (10 μM), 5 μL of KOD FX Neo Buffer, 2 μL of dNTPs (2 mM each), 0.2 μL of KOD FX Neo polymerase, and nuclease-free water. Thermal cycling consisted of an initial denaturation at 95 °C for 5 min, followed by 20 cycles of denaturation at 95 °C for 30 s, annealing at 50 °C for 30 s, and extension at 72 °C for 40 s, with a final extension at 72 °C for 7 min [30]. PCR products were purified and sequenced on an Illumina NovaSeq 6000 platform by Biomarker Technologies Co., Ltd. (Beijing, China).

### 2.4. Sequence Processing and Taxonomic Assignment

Raw sequencing reads were processed using QIIME 2 version 2019.4. Primer sequences were removed before quality filtering, denoising, paired-end merging, and chimera removal. Amplicon sequence variants (ASVs) were generated using the DADA2 pipeline [31], and singleton ASVs were removed before further analysis. Bacterial 16S rRNA gene ASVs were taxonomically assigned against the Greengenes reference database (Release 13.8), whereas fungal ITS ASVs were assigned against the UNITE reference database (Release 8.0), using the QIIME 2 classify-sklearn Naive Bayes classifier. The bacterial and fungal ASV tables were rarefied separately to a common sequencing depth before diversity and community analyses.

### 2.5. Statistical Analyses

α-diversity was calculated separately for bacterial and fungal communities using Observed ASVs, Chao1, Shannon, and Simpson indices. Differences among the eight site–compartment groups were tested using Kruskal–Wallis tests, followed by Dunn’s tests with Benjamini–Hochberg (BH) correction for multiple comparisons [32]. Rhizosphere and endosphere samples with the same site and replicate number were also compared within each sampling site using paired Wilcoxon signed-rank tests. *p* values from these paired comparisons were adjusted using the Benjamini–Hochberg method. Community composition was further examined using Bray–Curtis dissimilarities and visualized by principal coordinate analysis (PCoA). PERMANOVA was used to test the effects of sampling site, root compartment, and their interaction on bacterial and fungal community composition [33]. Homogeneity of multivariate dispersion was checked using the β-disper procedure following Anderson (2006) [34].

Taxonomic composition was summarized at the phylum and genus levels using relative abundance. The ten most abundant phyla and fifteen most abundant genera were displayed in stacked bar plots, and genus-level patterns were also shown using Z-score standardized heatmaps.

Changes in community membership between the rhizosphere and root compartment were examined using shared, stable, and core ASVs. Shared ASVs were defined as ASVs detected in both the rhizosphere and endosphere at the corresponding comparison level. Stable ASVs were defined as ASVs detected in at least two of the three replicate samples within each sampling site–compartment group. Following the prevalence–abundance concept commonly used in core microbiome studies [35], overall core ASVs were defined as taxa occurring in at least 75% of samples within a compartment and with a mean relative abundance of at least 0.01%. Site-specific core ASVs were defined as ASVs detected in at least two of the three replicate samples within each sampling site–compartment group and with a mean relative abundance of at least 0.01%. Shared and compartment-specific ASVs were compared between the rhizosphere and endosphere, and overlap between core communities was summarized using Jaccard similarity. We also calculated the proportion of rhizosphere stable ASVs that were detected as stable ASVs in the corresponding endosphere. This value was used as a descriptive rhizosphere-referenced stable-ASV overlap proportion.

Indicator taxa were identified separately for bacterial and fungal communities at the genus level. Genera present in at least 10% of samples, with a mean relative abundance of at least 0.01% and non-zero variance, were overlap for analysis. Compartment-associated taxa were identified using the IndVal.g statistic, with permutations restricted within matched site–replicate pairs to retain the paired sampling structure [36,37]. Site-associated taxa were tested separately within the rhizosphere and endosphere. *p* values were adjusted using the BH method, and FDR < 0.05 was used as the significance threshold [32].

Genus-level relative abundances were also compared between matched rhizosphere and endosphere samples using paired Wilcoxon signed-rank tests. Site-associated differences within each compartment were tested using Kruskal–Wallis tests, followed by Dunn tests with BH correction when the overall test was significant. Genera supported by both the indicator analysis and the paired abundance comparison in the same direction were considered high-confidence compartment-associated candidates.

Random Forest models were used to examine how well genus-level bacterial and fungal profiles distinguished rhizosphere from endosphere samples [38]. Relative abundances were transformed as log10(x + 10^−6^), and bacterial and fungal models were fitted separately using 2000 trees with the ranger implementation [39]. Model performance was evaluated using leave-one-pair-out cross-validation. In each round, the matched rhizosphere and endosphere samples with the same site and replicate number were left out together and used as the test set. Accuracy, balanced accuracy, sensitivity, specificity, and area under the ROC curve (AUC) were calculated. Permutation importance was used to rank genera, and the top 20 genera were treated as supporting classification evidence rather than as statistically significant taxa. Taxa with unclassified, ambiguous, or database placeholder names were retained in the Supplementary Tables but were not treated as formally resolved genera in the main candidate set.

Soil physicochemical data were used to describe the environmental background of the four sampling sites. Three independent rhizosphere soil samples were analyzed at each site, but these measurements were not matched one-to-one with the microbial samples. Soil variables were therefore treated as site-level environmental background and were not used as sample-level predictors in Mantel tests, constrained ordination, or related multivariate analyses.

All statistical analyses were performed in R version 4.5.1. The main analyses used the vegan, indicspecies, ranger, and ggplot2 packages. Unless otherwise stated, *p* values were adjusted using the Benjamini–Hochberg method, and adjusted *p* < 0.05 was considered statistically significant.

## 3. Results

### 3.1. Soil Physicochemical Background Differed Among Sampling Sites

Soil physicochemical properties differed among the four sampling sites (Figure S2; Table S2). Soil organic matter (SOM) and total nitrogen (TN) were highest at the Source of the Pearl River (ZJY), with mean values of 497.12 g/kg and 13.42 g/kg, respectively. Both variables were much lower at Huadianba, Cangshan Mountain (CS), where SOM was 78.90 g/kg and TN was 2.64 g/kg. Hydrolyzable nitrogen (HN) showed a similar pattern, ranging from 205.60 mg/kg at CS to 645.87 mg/kg at ZJY. Phosphorus and potassium showed different patterns across sites. Total phosphorus (TP) was highest at Junzi Mountain (JZ; 1.61 g/kg), whereas the lowest mean value occurred at ZJY (0.75 g/kg). Available phosphorus (AP) decreased from 75.95 mg/kg at JZ to 21.82 mg/kg at CS. Available potassium (AK) was relatively high at JZ and ZJY, with values of 368.10 and 386.87 mg/kg, respectively, but was lower at AL and CS. In contrast, total potassium (TK) was higher at the two higher-elevation sites, reaching 11.50 g/kg at Ailao Mountains (AL) and 13.88 g/kg at CS.

Soils at all four sites were acidic, with pH values ranging from 4.09 to 5.43. The lowest pH was recorded at ZJY, while CS had the highest mean pH. The four sampling sites showed distinct soil nutrient profiles rather than a consistent change in all soil properties along the elevation gradient.

### 3.2. α-Diversity Varied Across Sampling Sites and Root Compartments

**Figure 1:**
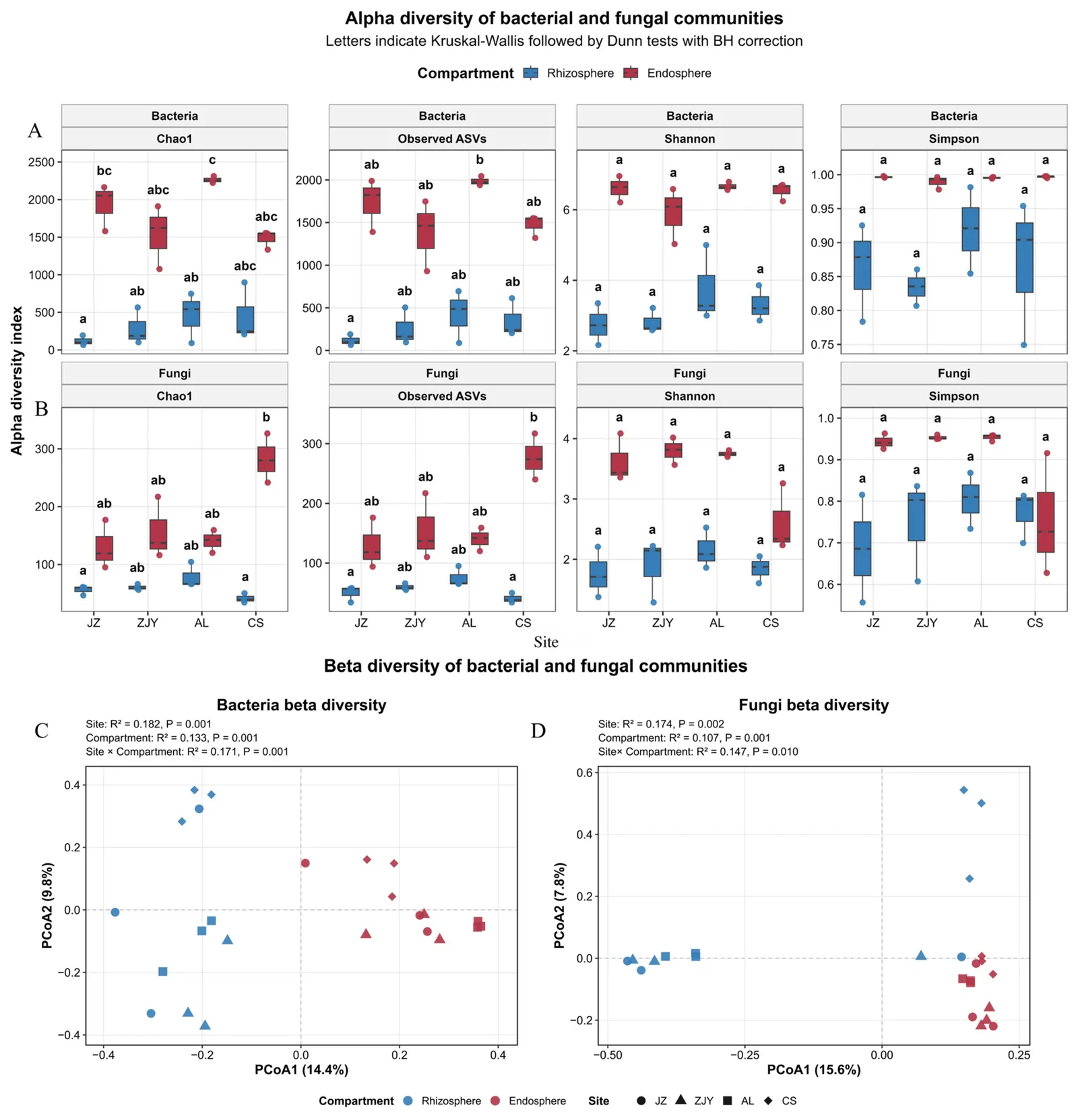
A- and β- diversity of bacterial and fungal communities across four sampling sites and root compartments. (**A**,**B**) α-diversity of bacterial (**A**) and fungal (**B**) communities based on Chao1, Observed ASVs, Shannon, and Simpson indices. Different lowercase letters indicate significant differences among site–compartment groups based on Kruskal–Wallis tests followed by Dunn’s tests with Benjamini–Hochberg correction (*p* < 0.05). (**C**,**D**) Bray–Curtis principal coordinate analysis (PCoA) of bacterial (**C**) and fungal (**D**) communities using all rhizosphere and endosphere samples. Colors indicate root compartment and point shapes indicate sampling site. PERMANOVA results show the associations with sampling site, compartment, and their interaction on community composition.

α-diversity of bacterial communities varied across sampling sites and between the rhizosphere and endosphere (Figure 1A). Differences were mainly observed for the richness indices. Chao1 and Observed ASVs were generally higher in the endosphere, with the highest values occurring at AL. Rhizosphere bacterial richness was lower, particularly at JZ, while ZJY, AL, and CS showed somewhat higher values. In contrast, Shannon and Simpson indices changed little among the sampling sites–compartment groups, and no clear differences were detected for these two indices.

Fungal α-diversity showed a similar pattern, with clearer differences in richness than in Shannon or Simpson diversity (Figure 1B). Chao1 and Observed ASVs were higher in the endosphere than in the rhizosphere at most sampling sites, with the highest richness found in the CS endosphere. Rhizosphere richness remained relatively low across the four sampling sites, especially at CS. Shannon and Simpson indices varied less, although the CS endosphere showed lower values and greater variation than the other endosphere groups.

The main differences in α-diversity were therefore associated with ASV richness, whereas Shannon and Simpson diversity remained relatively stable across most site–compartment combinations. However, paired comparisons within individual sampling sites did not detect significant rhizosphere–endosphere differences after BH correction (Figure S7).

### 3.3. Sampling Site and Root Compartment Were Associated with Differences in Microbial Community Composition

Bray–Curtis PCoA showed differences in bacterial and fungal community composition among sampling sites and between the rhizosphere and endosphere (Figure 1C,D). For bacteria, the first two PCoA axes explained 14.4% and 9.8% of the variation, respectively. PERMANOVA indicated significant associations with sampling site (R^2^ = 0.182, *p* = 0.001), compartment (R^2^ = 0.133, *p* = 0.001), and their interaction (R^2^ = 0.171, *p* = 0.001). For fungi, the first two axes explained 15.6% and 7.8% of the variation, respectively. Fungal community composition was also associated with sampling site (R^2^ = 0.174, *p* = 0.002), compartment (R^2^ = 0.107, *p* = 0.001), and their interaction (R^2^ = 0.147, *p* = 0.010). These results indicate that both sampling site and compartment were associated with variation in community composition, with the association between compartment and community composition differing among sites.

### 3.4. Taxonomic Composition Differed Between the Rhizosphere and Endosphere

**Figure 2:**
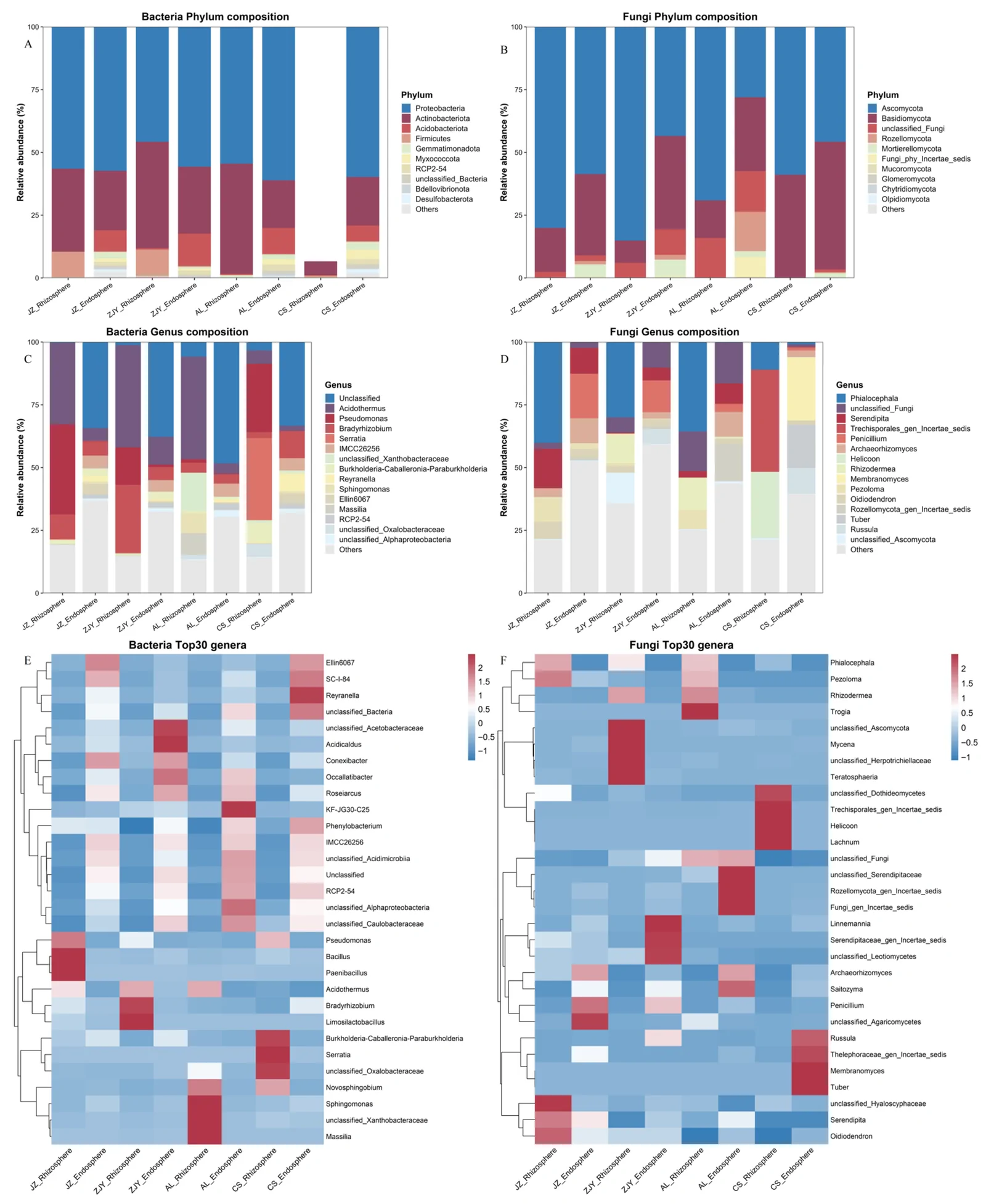
Taxonomic composition of bacterial and fungal communities in the rhizosphere and endosphere across four sampling sites. (**A**,**B**) Relative abundance of bacterial and fungal communities at the phylum level. (**C**,**D**) Relative abundance of bacterial and fungal communities at the genus level. (**E**,**F**) Heatmaps showing the top 30 bacterial and fungal genera across sampling sites and compartments. Colors in the heatmaps represent standardized relative abundance (Z-score), with red indicating higher and blue indicating lower values. JZ, Junzi Mountain; ZJY, Source of the Pearl River; AL, Ailao Mountains; CS, Huadianba, Cangshan Mountain.

Taxonomic composition varied among sampling sites and between the rhizosphere and endosphere (Figure 2). At the phylum level, bacterial communities were mainly composed of Proteobacteria and Actinobacteriota (Figure 2A). Proteobacteria was the dominant phylum in most samples and accounted for a particularly large proportion in the CS rhizosphere. Actinobacteriota also represented a major fraction of the bacterial communities in several JZ, ZJY, and AL samples. Other phyla, including Acidobacteriota and Firmicutes, occurred at lower relative abundances and were more apparent in some endosphere samples. Fungal communities were mainly dominated by Ascomycota and Basidiomycota (Figure 2B). Their relative proportions differed among sampling sites and compartments. Ascomycota was particularly abundant in the ZJY rhizosphere, while the fungal composition of AL and CS endosphere samples was more mixed, with larger contributions from Basidiomycota and several less abundant phyla.

Differences became more apparent at the genus level (Figure 2C,D). Bacterial communities contained several abundant genera, including *Acidothermus*, *Pseudomonas*, *Bradyrhizobium*, and *Serratia*, together with a relatively large proportion of unclassified taxa. Their relative abundance varied across sampling sites and compartments. The Top30 heatmap further showed distinct distribution patterns (Figure 2E). *Pseudomonas*, *Bacillus*, and *Paenibacillus* had relatively high standardized abundance in the JZ rhizosphere, whereas *Bradyrhizobium* and *Limosilactobacillus* were more abundant in the ZJY rhizosphere. *Sphingomonas* and *Massilia* were more prominent in the AL rhizosphere, while *Serratia* and *Burkholderia-Caballeronia-Paraburkholderia* showed higher values in the CS rhizosphere.

Fungal genera also showed clear variation among the sample groups (Figure 2D,F). *Phialocephala*, *Penicillium*, *Archaeorhizomyces*, and several unclassified fungal groups were among the more abundant taxa. The heatmap showed that some genera were mainly associated with particular site–compartment combinations. For example, *Serendipita* and *Oidiodendron* showed relatively high standardized abundance in the JZ rhizosphere, while *Mycena* and *Teratosphaeria* were higher in the ZJY rhizosphere. *Trogia* was more abundant in the AL rhizosphere, whereas *Russula*, Thelephoraceae-related taxa, *Membranomyces*, and *Tuber* showed higher values in the CS endosphere.

Phylum-level communities were dominated by a few major taxa, whereas genus-level profiles showed stronger variation among sampling sites and between the rhizosphere and endosphere.

### 3.5. Stable and Core ASV Overlap Differed Between the Rhizosphere and Endosphere

**Figure 3:**
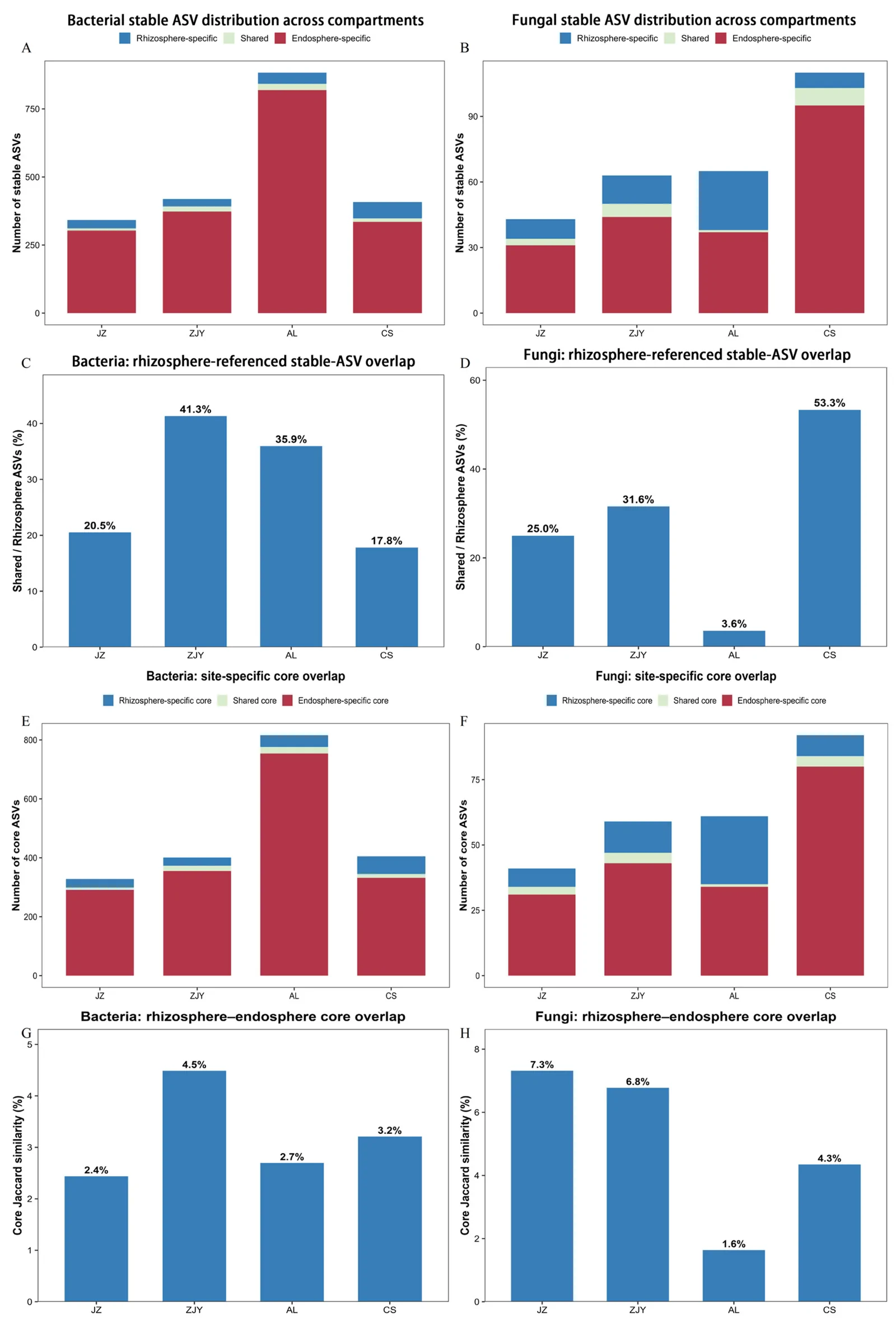
Compartment-associated turnover of stable and site-specific core ASVs across four sampling sites. (**A**,**B**) Numbers of rhizosphere-specific, shared, and endosphere-specific stable ASVs for bacterial (**A**) and fungal (**B**) communities. (**C**,**D**) Overlap proportion of rhizosphere stable ASVs in the endosphere for bacteria (**C**) and fungi (**D**), calculated as the number of shared stable ASVs divided by the number of rhizosphere stable ASVs × 100%. (**E**,**F**) Numbers of rhizosphere-specific core, shared core, and endosphere-specific core ASVs for bacteria (**E**) and fungi (**F**). (**G**,**H**) Jaccard similarity between rhizosphere and endosphere site-specific core ASV sets for bacteria (**G**) and fungi (**H**). Stable ASVs were defined as ASVs detected in at least two of the three replicates within each Site × Compartment group. Site-specific core ASVs were detected in at least two of the three replicates and had a mean relative abundance ≥ 0.01%.

Stable ASV composition differed between the rhizosphere and endosphere across the four sampling sites (Figure 3A–D; Table S3). For bacteria, JZ contained 31 rhizosphere-specific, 8 shared, and 303 endosphere-specific stable ASVs, with 20.5% of the rhizosphere stable ASVs also detected as stable ASVs in the endosphere. At ZJY, 27 ASVs were rhizosphere-specific, 19 were shared, and 373 were endosphere-specific, giving the highest rhizosphere-referenced bacterial stable-ASV overlap proportion of 41.3%. AL had 41 rhizosphere-specific, 23 shared, and 819 endosphere-specific ASVs, with a rhizosphere-referenced stable-ASV overlap proportion of 35.9%. At CS, 60 ASVs were specific to the rhizosphere, 13 were shared, and 335 were specific to the endosphere. The overlap proportion was 17.8%, the lowest among the sampling sites.

Fungal stable ASVs showed a different pattern (Figure 3B,D; Table S3). JZ had 9 rhizosphere-specific, 3 shared, and 31 endosphere-specific ASVs, with a overlap proportion of 25.0%. At ZJY, the corresponding numbers were 13, 6, and 44, and overlap reached 31.6%. AL showed the lowest fungal overlap, at 3.6%, with only one shared ASV, compared with 27 rhizosphere-specific and 37 endosphere-specific ASVs. In contrast, CS had 7 rhizosphere-specific, 8 shared, and 95 endosphere-specific ASVs, with the highest fungal overlap proportion of 53.3%. ASV intersections among sampling sites within the rhizosphere and endosphere are shown in Figure S4.

The overall core microbiome showed little overlap between compartments (Table S4). When the core was defined across all 12 samples within each compartment, 6 bacterial ASVs met the rhizosphere core criteria and 33 met the endosphere core criteria. Three ASVs met the core criteria in both compartments. For fungi, only 1 ASV was identified as a rhizosphere core member and 3 ASVs as endosphere core members. No fungal ASV simultaneously met the core criteria in both compartments.

Site-specific core communities also showed limited overlap between the rhizosphere and endosphere (Figure 3E–H; Table S5). The distribution of individual site-specific core ASVs across sampling sites and compartments is shown in Figure S3. Bacterial rhizosphere core size increased from 37 ASVs at JZ to 46 at ZJY, 62 at AL, and 73 at CS. Endosphere core communities were larger at all sampling sites, with 299, 373, 776, and 345 ASVs, respectively. The number of shared core ASVs remained small, ranging from 8 at JZ to 22 at AL. Jaccard similarity was highest at ZJY (4.5%), followed by CS (3.2%), AL (2.7%), and JZ (2.4%) (Figure 3E,G; Table S5).

Fungal site-specific core communities contained fewer ASVs. The rhizosphere core included 10 ASVs at JZ, 16 at ZJY, 27 at AL, and 12 at CS, whereas the corresponding endosphere cores contained 34, 47, 35, and 84 ASVs. Only 1–4 core ASVs were shared between compartments at each sampling site. Jaccard similarity was relatively high at JZ (7.3%) and ZJY (6.8%), decreased to 4.3% at CS, and was lowest at AL (1.6%) (Figure 3F,H; Table S5).

Across the stable and core ASV sets, only a limited fraction was shared between the rhizosphere and endosphere. The degree of overlap varied among sampling sites, and bacteria and fungi did not show the same site-level pattern.

### 3.6. Indicator Analyses Identified Consistent Compartment-Associated Genera

At the genus level, 177 bacterial and 114 fungal genera were included in the compartment-association analysis (Table S6). Indicator value analysis identified 74 bacterial and 11 fungal genera associated with either the rhizosphere or endosphere, while the paired Wilcoxon test detected 71 bacterial and 12 fungal genera with significant abundance differences between the two compartments (FDR < 0.05; Table S6). Most of the indicator genera were associated with the endosphere, especially for bacteria (Figure S5).

Combining the IndVal and paired Wilcoxon results reduced the candidate set to 79 genera with consistent support from both methods, including 68 bacterial and 11 fungal genera (Table S7). Among the bacterial candidates, *Acidothermus* and *Pseudomonas* were associated with the rhizosphere, whereas many of the remaining genera were associated with the endosphere. For fungi, *Phialocephala* was the main rhizosphere-associated genus, while *Linnemannia*, *Metapochonia*, *Mortierella*, *Penicillium*, *Podila*, *Saitozyma*, and *Trichoderma* were associated with the endosphere (Table S7; Figure S5).

**Figure 4:**
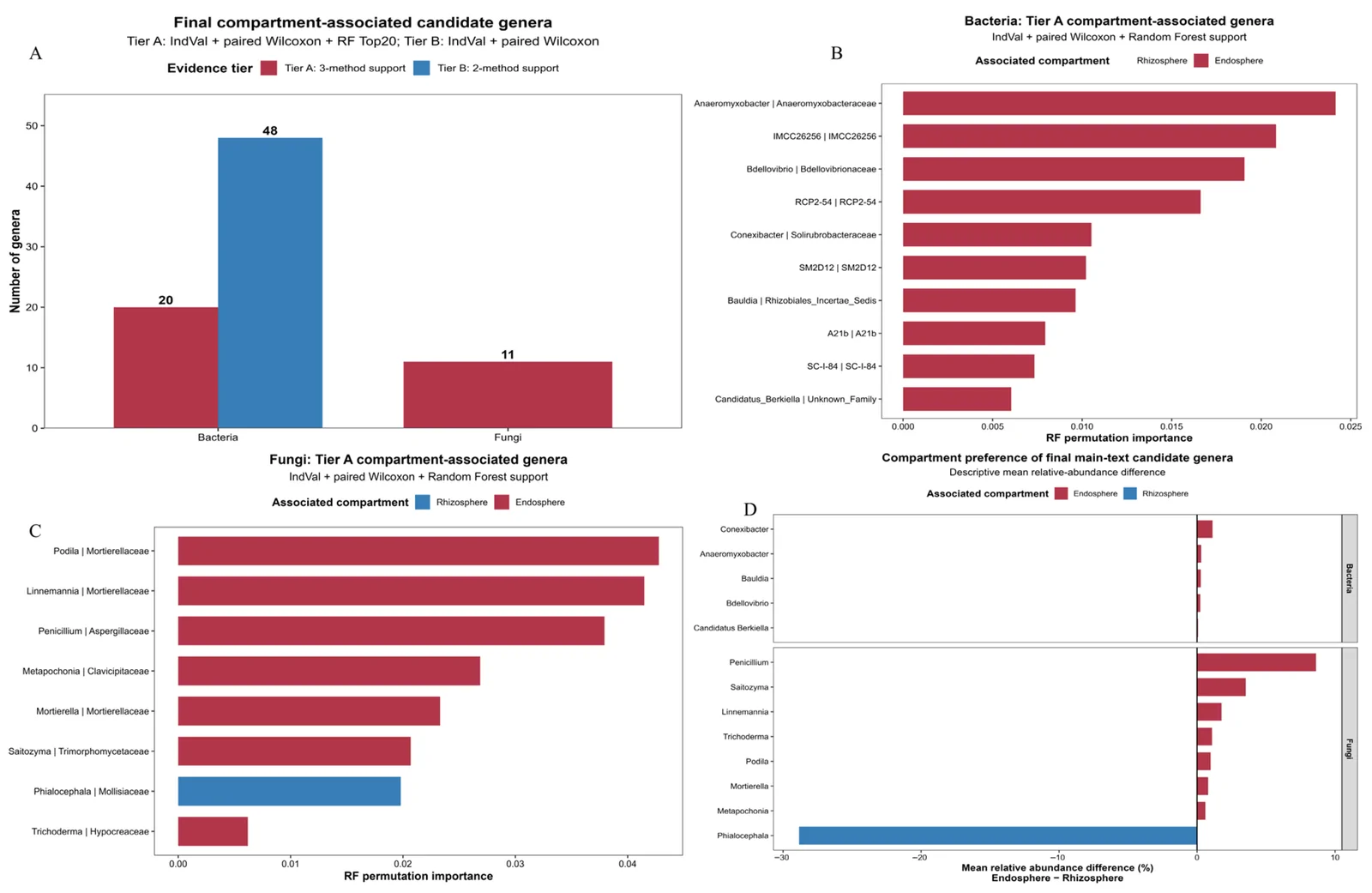
Compartment-associated bacterial and fungal genera supported by multiple analytical approaches. (**A**) Numbers of bacterial and fungal candidate genera according to evidence tier. Tier A candidates were supported by IndVal, paired Wilcoxon analysis, and Random Forest Top-20 permutation importance, whereas Tier B candidates were supported by IndVal and paired Wilcoxon analysis. (**B**,**C**) Random Forest permutation importance of selected Tier A bacterial (**B**) and fungal (**C**) genera. Bar colors indicate the associated compartment. (**D**) Mean relative abundance differences between the endosphere and rhizosphere for the taxonomically interpretable genera overlap for the main text. Positive values indicate higher mean abundance in the endosphere, whereas negative values indicate higher mean abundance in the rhizosphere. Random Forest permutation importance was used as supporting classification evidence rather than as a significance test.

Random Forest analysis was then used as an additional line of support for the dual-method candidates. Twenty bacterial genera were supported by all three methods, while another 48 overlap support by IndVal and the paired Wilcoxon test only (Figure 4A). All 11 fungal candidates were also supported by Random Forest, giving 11 three-method fungal candidates (Figure 4A; Table S8). The LOPO cross-validated Random Forest models separated rhizosphere and endosphere samples well, with an AUC of 1.000 for both bacterial and fungal communities (Figure S6).

Among the final bacterial candidates, *Anaeromyxobacter*, *Bdellovibrio*, *Conexibacter*, *Bauldia*, and *Candidatus berkiella* were overlap as taxonomically interpretable genera for the main text (Figure 4B; Table S8). These genera were associated with the endosphere. Their mean relative abundances were also higher in the endosphere than in the rhizosphere, although the size of the difference varied among genera (Figure 4D; Table S8).

The fungal candidates showed a broader range of abundance differences between compartments (Figure 4C,D). *Podila*, *Linnemannia*, *Penicillium*, *Metapochonia*, *Mortierella*, *Saitozyma*, and *Trichoderma* were associated with the endosphere, whereas *Phialocephala* was associated with the rhizosphere. The difference was particularly large for *Phialocephala*, with a mean relative abundance of 29.15% in the rhizosphere and 0.31% in the endosphere. *Penicillium* showed the opposite pattern, increasing from 0.004% in the rhizosphere to 8.63% in the endosphere (Table S8).

The agreement among IndVal, paired abundance tests, and Random Forest support narrowed the initial genus set to a smaller group of compartment-associated candidates. These candidates differed between bacteria and fungi and were mainly associated with the endosphere, with a few clear rhizosphere-associated genera remaining.

## 4. Discussion

Root-associated bacterial and fungal communities of *Rhododendron yunnanense* differed both among sampling sites and between the rhizosphere and endosphere. In our study, differences in α-diversity were mainly reflected in ASV richness, whereas Shannon and Simpson diversity varied less across most site–compartment combinations. Similar compartment-associated differentiation has been reported in other plant root systems, where the rhizosphere and root endosphere harbor distinct microbial assemblages [40].β- diversity analysis further showed that sampling site, root compartment, and their interaction were all associated with variation in community composition, suggesting that compartment-related patterns were not identical across sampling sites. This is consistent with recent evidence that root-associated microbial communities can respond strongly to changes in the surrounding soil environment [41]. In addition, the limited overlap of stable and core ASVs between the rhizosphere and endosphere showed that substantial community turnover occurred across the two compartments. The particularly low overlap observed for fungi is also consistent with recent work showing clear differentiation between rhizospheric and root-endospheric fungal communities [42].

The higher ASV richness observed in the endosphere should not be interpreted as a general increase in microbial diversity during root colonization. Changes in α-diversity across root compartments are not always consistent among plant species or microbial groups. For example, bacterial phylogenetic diversity was higher in the endosphere than in the rhizosphere of Dendrobium huoshanense, whereas other plant systems have shown lower bacterial and fungal richness in the endosphere [43,44]. In *R. yunnanense*, the clearer differences in Chao1 and Observed ASVs, together with the relatively small changes in Shannon and Simpson indices, suggest that compartment-associated variation was mainly reflected in the number of detected ASVs rather than in major changes in community evenness. At the same time, the significant associations of sampling site, compartment, and their interaction with community composition indicate that rhizosphere–endosphere differentiation was not uniform among sampling sites. Variation among defined root-associated niches has also been reported at much smaller spatial scales, suggesting that root microbiome structure can differ even within apparently similar root environments [45]. These results therefore point to a context-dependent pattern of compartment differentiation rather than a simple directional change in microbial diversity from the rhizosphere to the root interior.

Beyond the diversity patterns, the limited overlap of stable and core ASVs provided further evidence of microbial turnover between the rhizosphere and endosphere. In *R. yunnanense*, most stable ASVs were restricted to one compartment, and the overlap of site-specific core communities was generally low. Under the predefined core criteria, only a small number of bacterial ASVs were shared between the two compartments, while no fungal ASV met the core threshold in both the rhizosphere and endosphere. Similar compartment-specific patterns have been reported in other plant systems. In grafted apple, only a small fraction of bacterial ASVs was shared between the rhizosphere and root endosphere [46], while clear differences in microbial assemblages and compartment-specific taxa were also observed in *Sedum alfredii* [47]. Studies including both bacterial and fungal communities have likewise shown substantial changes in microbial membership along the soil–root continuum [44,48]. These observations suggest that taxa consistently detected in the rhizosphere were not necessarily detected at comparable prevalence or abundance in the root compartment. This distinction is particularly relevant when defining a core microbiome, because core membership depends not only on the presence of a taxon but also on its consistency across samples [49]. In our study, the absence of a shared fungal core therefore does not imply that no fungal ASVs occurred in both compartments. Rather, the shared fungi did not meet the prevalence and abundance thresholds required to qualify as core members in both niches. The variation in stable-ASV overlap among sampling sites further suggests that overlap turnover was context dependent. Recent work along soil–root continua also shows that microbial overlap can vary with host and environmental context rather than following a fixed pattern [50].

Indicator analyses further narrowed the compartment-associated signal to a smaller set of genera with consistent support across methods. Combining IndVal with paired abundance testing, and then using Random Forest as an additional line of classification support, reduced the dependence of candidate selection on a single analytical criterion. Among the taxonomically interpretable bacterial genera overlap for the main text, *Anaeromyxobacter*, *Bdellovibrio*, *Conexibacter*, *Bauldia*, and *Candidatus berkiella* were associated with the endosphere. Some of these genera have also been reported from root-associated communities in other plants. *Anaeromyxobacter*, for example, was recently identified as an important member of the rice root microbiome under altered oxygen conditions, and a representative strain improved rice submergence tolerance [51]. *Conexibacter* has also been reported as enriched in root tissues of *Panax quinquefolius* [52]. Fungal candidates showed a similar tendency toward the endosphere, including *Podila*, *Linnemannia*, *Penicillium, Metapochonia, Mortierella, Saitozyma*, and *Trichoderma*, whereas *Phialocephala* was strongly associated with the rhizosphere. Penicillium has been detected in root endosphere communities of other plant species, and endophytic Trichoderma strains have also been isolated from plant roots [44,53]. The rhizosphere association of *Phialocephala* in *R. yunnanense* is of particular interest because members of this genus include dark septate endophytes that are well known from woody plant roots [54,55]. This contrast also suggests that compartment preference should not be assumed to be fixed at the genus level. Root-colonizing fungal lifestyles can vary with host identity and environmental context [56]. Therefore, the genera identified here are best regarded as compartment-associated candidates rather than functionally defined members of the *R. yunnanense* microbiome. Their ecological roles will require direct validation using cultured isolates or other functional approaches.

Site-associated differences in microbial communities may also be related to variation in the local environmental background. The four sampling sites in this study differed in several soil physicochemical properties, including pH and nutrient-related variables. Similar patterns have been reported in alpine *Rhododendron nitidulum* shrub ecosystems, where bacterial and fungal communities varied among elevations and were associated with soil water content, pH, nitrate, available phosphorus, and root traits [19]. Environmental associations are not restricted to *Rhododendron*. Across broad-leaved forest sites in Europe, variation in root and soil microbiomes was related to a combination of climate, soil properties, vegetation, and root symbioses rather than to a single environmental variable [57]. Experimental studies have also shown that root-associated microbiomes can respond to changes in soil nutrient conditions and water availability, although the magnitude and direction of these responses depend on the host and environmental setting [41,58]. In the present study, soil physicochemical properties were measured using three independent rhizosphere soil samples per site, but these measurements were not matched to individual microbial samples. They were therefore used to characterize site-level environmental background rather than as sample-level predictors of microbial composition. Accordingly, the observed soil differences are better regarded as part of the environmental context of each sampling site rather than as evidence that any individual soil variable, such as pH or nutrient availability, directly determined the microbial patterns. The site-associated differences observed here may instead reflect the combined influence of edaphic conditions and other local factors that could not be separated with the present sampling design.

These findings should be interpreted with several constraints in mind. First, each environmental setting was represented by a single geographic sampling site. Therefore, the observed site-associated differences cannot be separated into independent effects of soil properties, climate, surrounding vegetation, geographic location, or other local factors. The geographic factor should therefore be interpreted as sampling-site identity rather than as an independently replicated sampling site treatment. In addition, each site–compartment group contained only three biological replicates, which limited the statistical power of within-site comparisons and may have reduced the ability to detect some rhizosphere–endosphere differences. Soil physicochemical properties were measured using three independent soil samples per site, but these measurements were not matched to each microbial sample. They were therefore used to describe site-level environmental background rather than to identify sample-level edaphic drivers of microbial variation. The use of 16S rRNA gene and ITS amplicon sequencing also limits interpretation mainly to taxonomic composition and relative abundance, with little direct information on microbial function or absolute abundance [59,60]. Although the Random Forest models showed high classification performance under leave-one-pair-out cross-validation, this performance was evaluated within the present dataset. Microbiome-based machine-learning models can be sensitive to sample size and dataset structure, and their ability to generalize should therefore be assessed with independent samples [61]. Future studies with larger sample sizes, additional geographically replicated sites, and matched environmental measurements would help clarify the site-associated patterns observed here. Isolation and inoculation experiments, together with approaches that directly measure microbial functional potential or activity, will also be needed to determine whether the compartment-associated genera identified in this study have consistent ecological roles in *R. yunnanense*.

## 5. Conclusions

This study showed that the root-associated microbiome of *Rhododendron yunnanense* differed across sampling sites and between the rhizosphere and endosphere. Differences in alpha diversity were mainly reflected in ASV richness, whereas Shannon and Simpson diversity varied less across site–compartment combinations. Community composition was associated with sampling site, root compartment, and their interaction in both bacterial and fungal communities.

Stable and core ASV analyses further revealed limited overlap between the rhizosphere and endosphere, indicating substantial turnover in microbial membership across root compartments. This pattern varied among sampling sites and differed between bacteria and fungi. By combining indicator-value analysis, paired abundance testing, and Random Forest classification, we further identified a smaller set of genera consistently associated with either the rhizosphere or endosphere.

Together, these findings indicate that both sampling site and root compartment were associated with variation in the *R. yunnanense* root microbiome. The low overlap of stable and core taxa highlights the distinct microbial composition of the rhizosphere and endosphere. The compartment-associated genera identified here provide candidates for future isolation and inoculation experiments, but their ecological roles in *R. yunnanense* remain to be tested directly.

## Data Availability

The raw amplicon sequencing reads of the bacterial 16S rRNA gene (V3–V4 region) and fungal ITS1 region generated from the rhizosphere soil and root endosphere of *Rhododendron yunnanense* have been deposited in the NCBI Sequence Read Archive (SRA) under BioProject accession number PRJNA1532524 (https://www.ncbi.nlm.nih.gov/bioproject/PRJNA1532524).

## Supplementary Materials

The following supporting information can be downloaded at https://www.mdpi.com/article/10.3390/microorganisms14102264/s1, Figure S1: Geographic distribution of sampling sites and schematic of rhizosphere and endosphere sampling; Figure S2: Soil physicochemical properties across the four sampling sampling sites; Figure S3: Distribution of site-specific core bacterial and fungal ASVs across sampling sites and root compartments; Figure S4: Sampling site-level overlap of bacterial and fungal ASVs in the rhizosphere and endosphere; Figure S5: Compartment-associated bacterial and fungal indicator genera identified by indicator value analysis; Figure S6: Leave-one-pair-out cross-validated ROC curves for bacterial and fungal Random Forest classification; Figure S7: Paired comparisons of bacterial and fungal alpha diversity between the rhizosphere and endosphere within each sampling site; Table S1: Sampling site information and sampling site characteristics; Table S2: Soil physicochemical properties of the four *Rhododendron yunnanense* sampling sampling sites; Table S3: Stable ASV sharing and overlap between the rhizosphere and endosphere across site; Table S4: Overall core ASV overlap between rhizosphere and endosphere bacterial and fungal communities; Table S5: Site-specific core ASV overlap and Jaccard similarity between the rhizosphere and endosphere; Table S6: Complete genus-level compartment association results from indicator value and paired abundance analyses; Table S7: High-confidence compartment-associated genera supported by both indicator value and paired abundance analyses; Table S8: Random Forest-supported compartment-associated candidate genera with taxonomic name quality control.
