# Supplementary material for: Site- and Compartment-Associated Differentiation of Root Microbiomes in Rhododendron yunnanense
Source: Microorganisms. 2026 Oct 5;14(10):2264. doi: 10.3390/microorganisms14102264 (PMC13707437; doi:10.3390/microorganisms14102264)
Supplement: Supplementary file 1 [file microorganisms-14-02264-s001.zip › microorganisms-4585127-supplementary.pdf]

Supplementary Information

Supplementary Figures

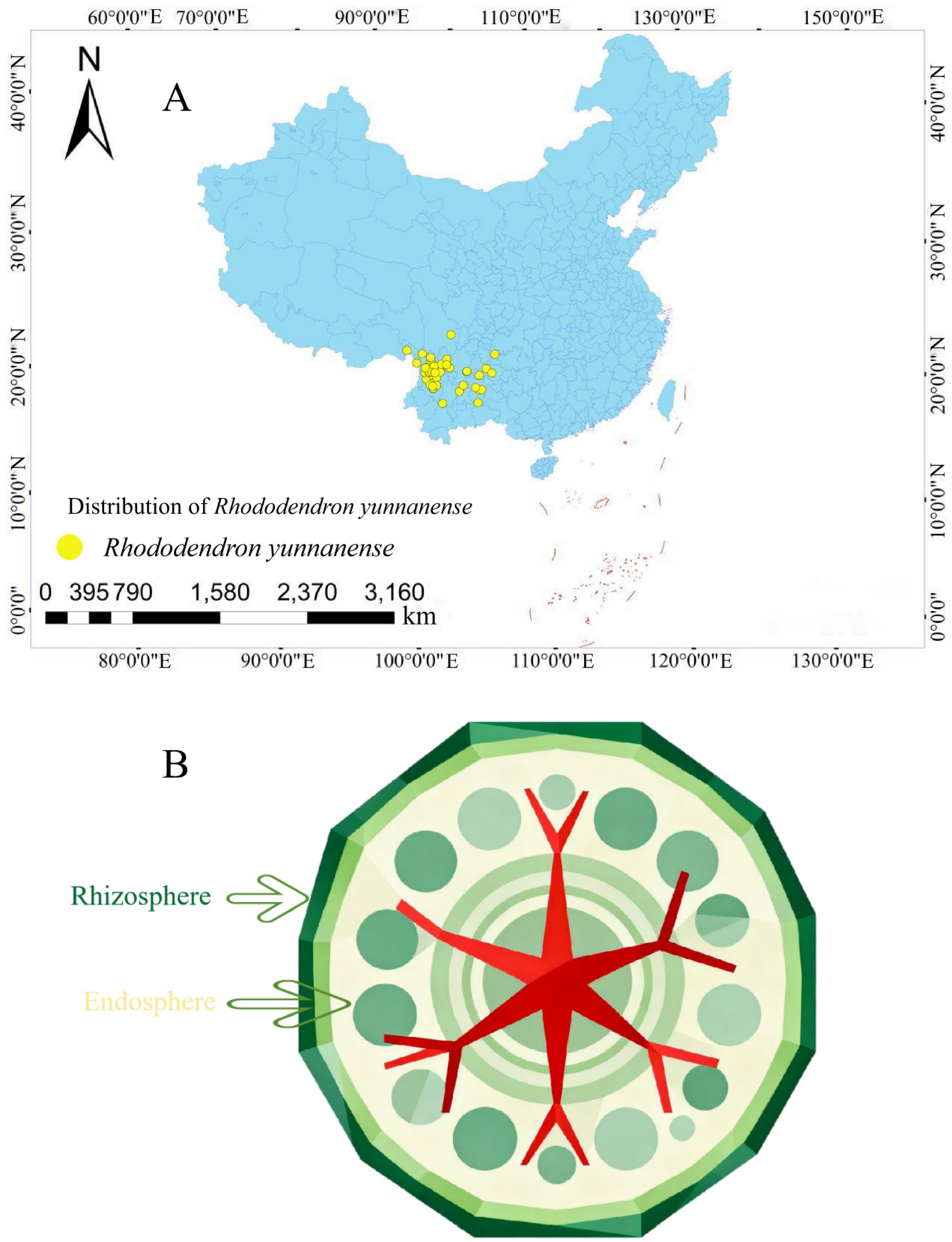

Figure S1. Geographic distribution of sampling sites and schematic illustration of rhizosphere and endosphere sampling. (A) Spatial distribution of sampling sites in

China; yellow dots indicate sampling locations.(B) Schematic cross-section of the root showing the two microbial niches sampled: the rhizosphere and the endosphere.

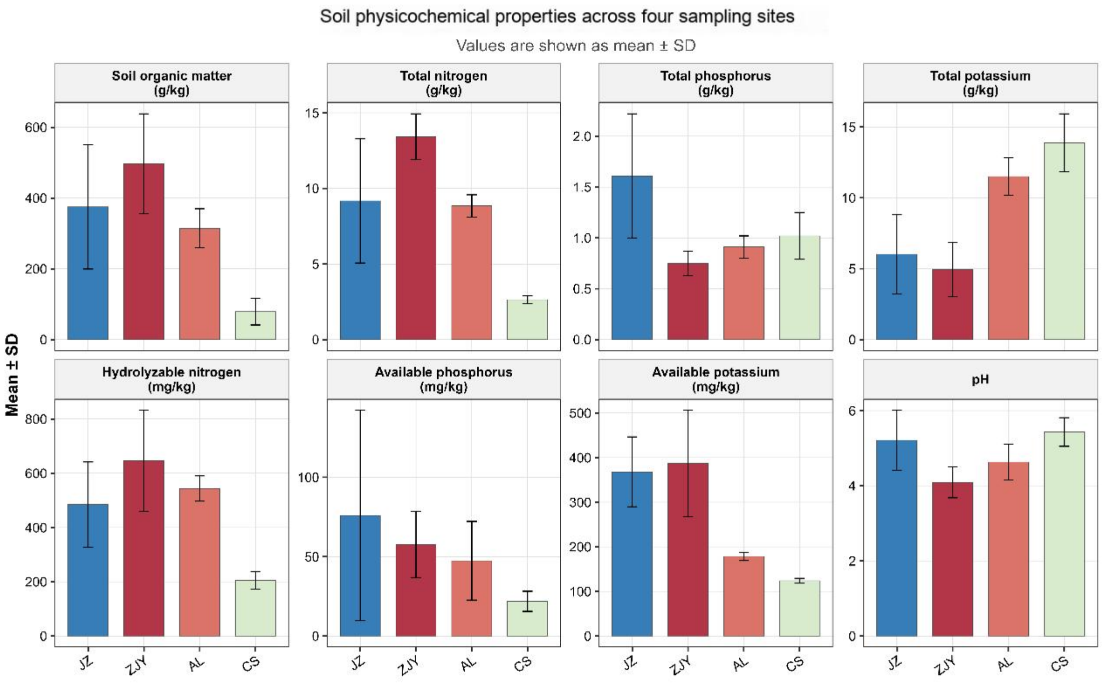

Figure S2.Soil physicochemical properties across the four sampling sites.Values are shown as mean ± SD.

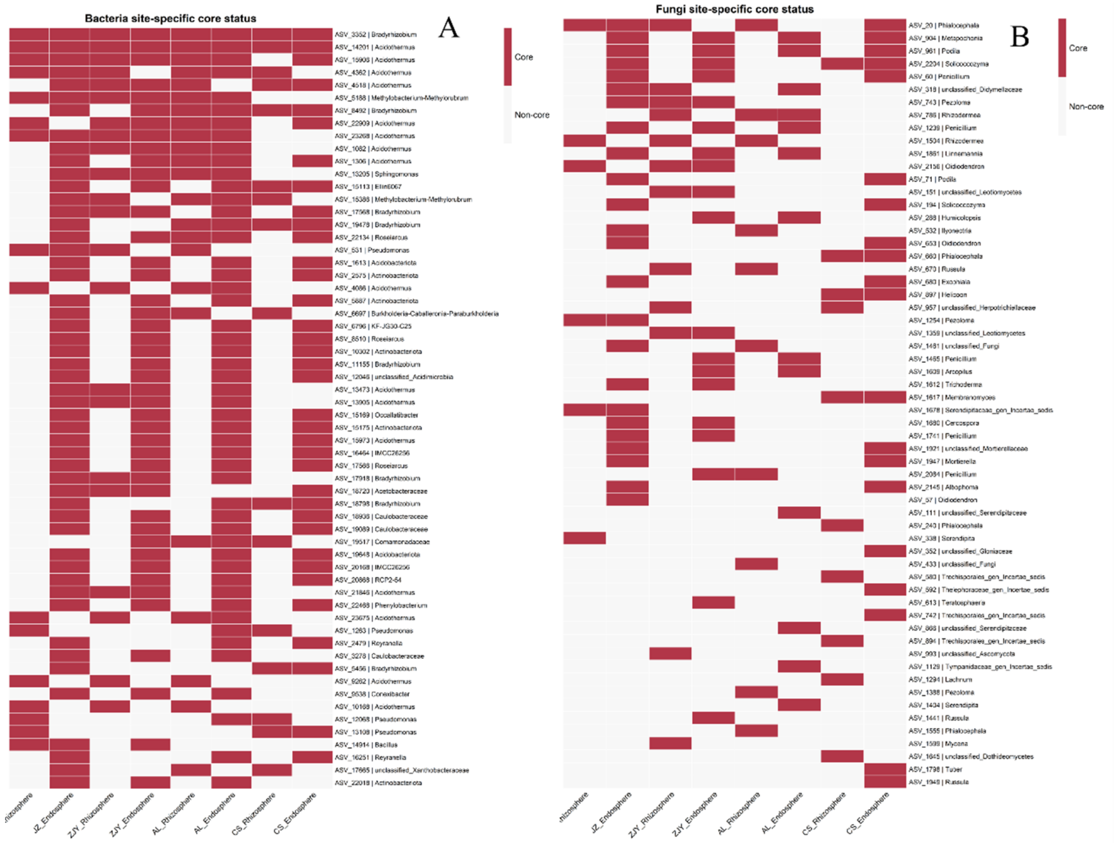

Fig. S3. Distribution of site-specific core ASVs across sampling sites and root compartments.(A) Bacterial and (B) fungal site-specific core ASVs in the rhizosphere and endosphere of the four sampling sites. Red cells indicate ASVs classified as site-specific core members in the corresponding site–compartment group, whereas white cells indicate non-core status. Taxonomic assignments are shown for each ASV.

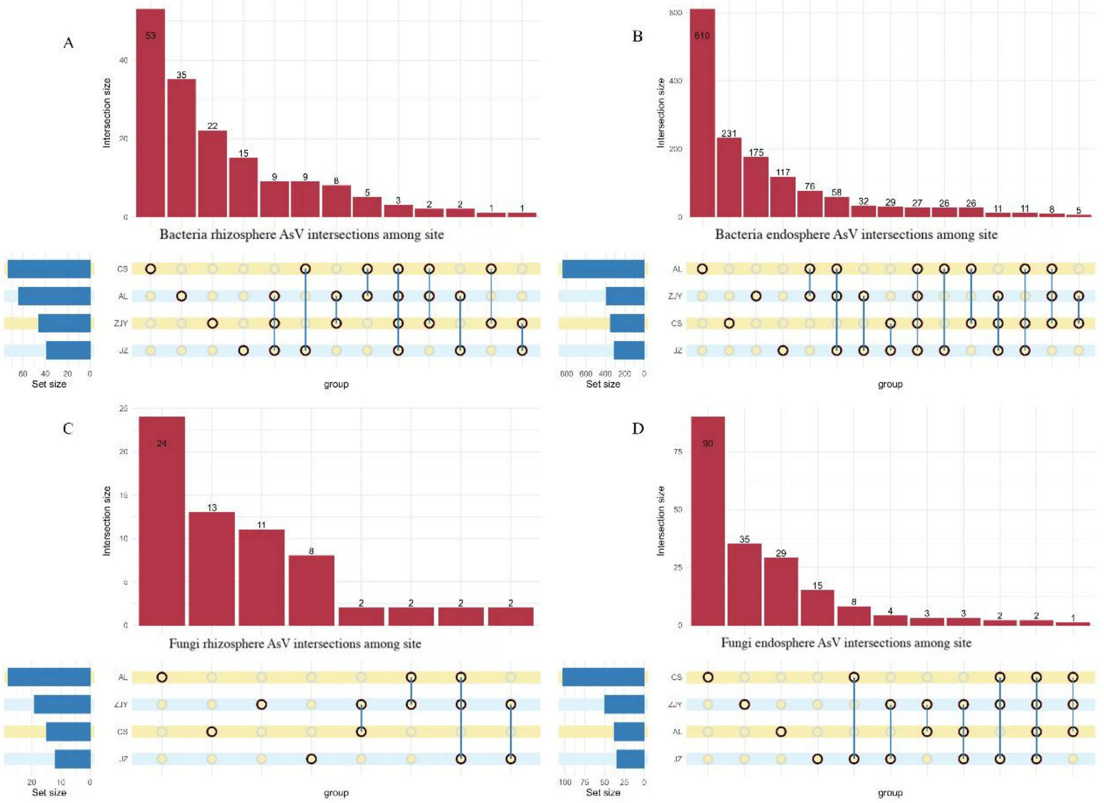

Fig. S4. Site-level overlap of bacterial and fungal ASVs in the rhizosphere and endosphere. UpSet plots show ASV intersections among the four sampling sites for (A) rhizosphere bacteria, (B) endosphere bacteria, (C) rhizosphere fungi, and (D) endosphere fungi. Vertical bars show intersection sizes, horizontal bars show the total ASV number for each sampling site, and connected dots indicate the sampling site combinations included in each intersection.

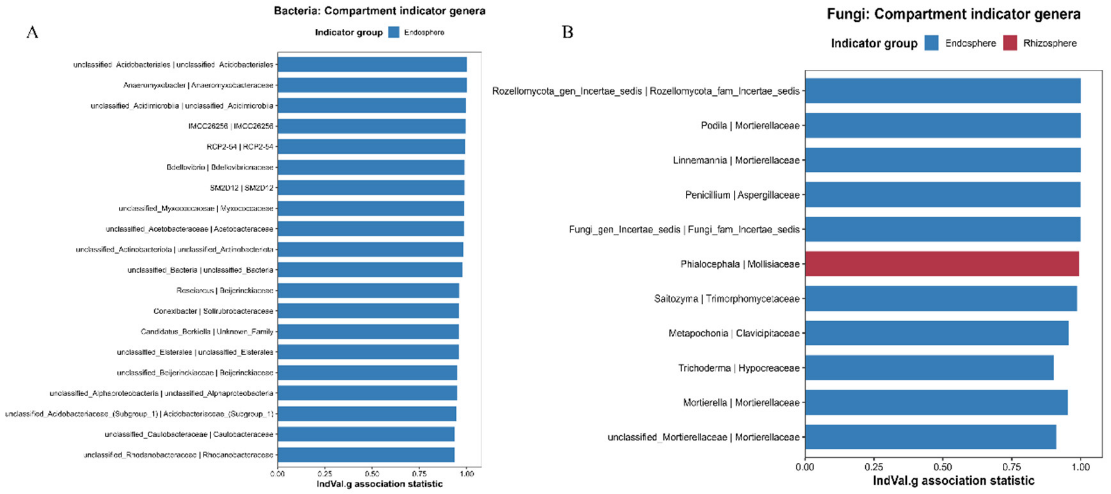

Fig. S5. Compartment indicator genera identified in bacterial and fungal communities.(A) Selected bacterial and (B) fungal genera associated with the rhizosphere or endosphere based on indicator value analysis. Bar length represents the IndVal.g statistic, and colors indicate the associated compartment. Genera shown met the IndVal significance criterion after FDR correction.

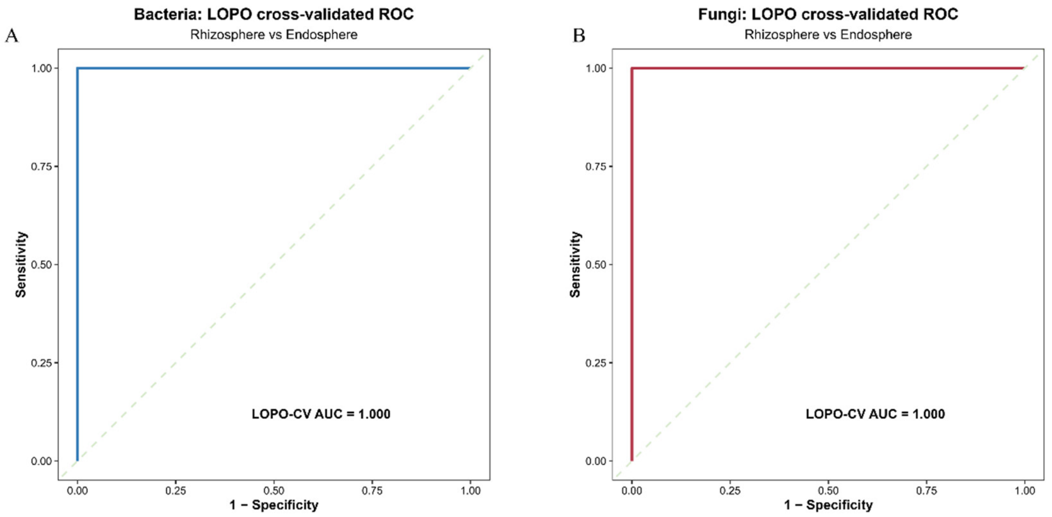

Fig. S6. Leave-one-pair-out cross-validated ROC curves for bacterial and fungal compartment classification.ROC curves are shown for (A) bacterial and (B) fungal Random Forest models used to distinguish rhizosphere and endosphere samples. Model performance was evaluated using leave-one-pair-out cross-validation (LOPO-CV). The area under the ROC curve was 1.000 for both bacterial and fungal models.

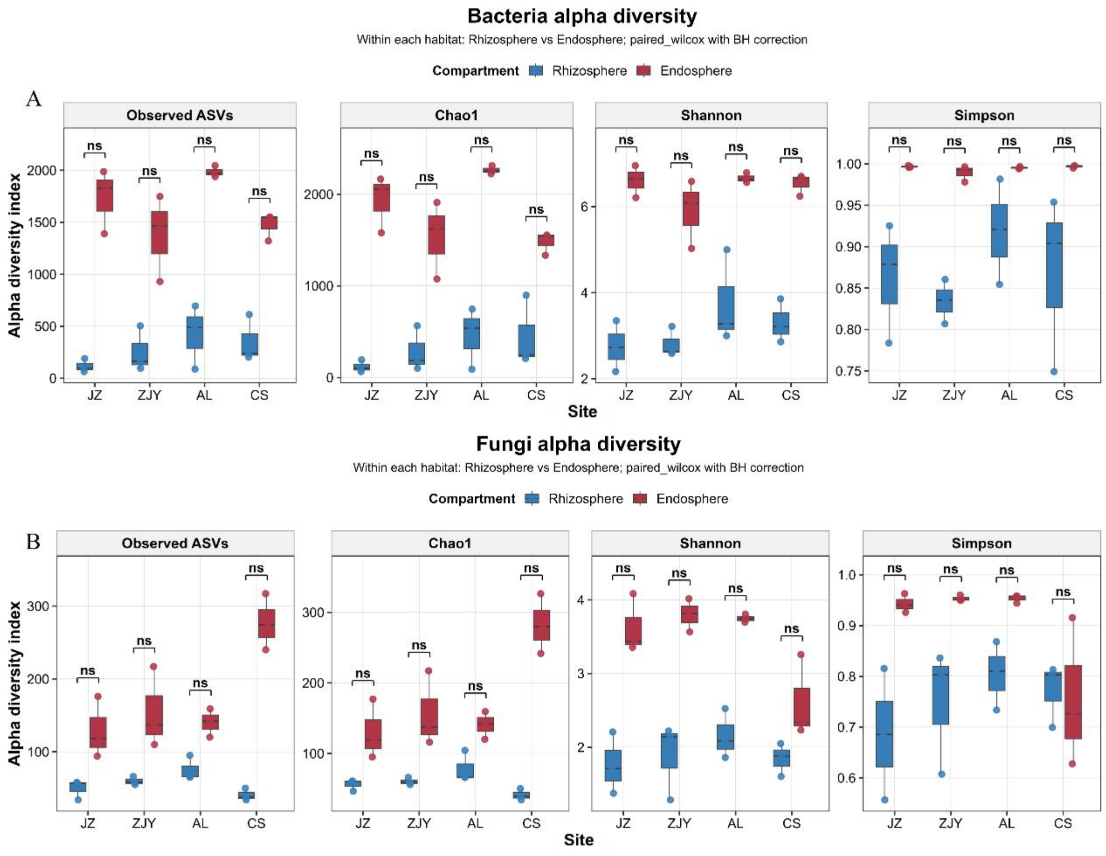

Figure S7. Paired comparisons of bacterial (A) and fungal (B) alpha diversity between rhizosphere and endosphere samples within each sampling site. Observed ASVs, Chao1, Shannon, and Simpson indices were compared using paired Wilcoxon signed-rank tests. *P* values were adjusted using the Benjamini–Hochberg method. ns indicates no significant difference after multiple-testing correction.

Supplementary Table

Table S1. Plot Information

Site code	Sampling code	City	Elevation	Latitude and longitude	Distribution pattern	Dominant tree species
J	Junzi Mountain	Shizong, Qujing	2252 m	E104°9′49.3″，N24°38′22.9″	Scattered distribution	*Rhododendron delavayi*、*Quercus aliena* var. *acutiserrata*）
Z	Zhujiangyuan	Zhanyi, Qujing	2305 m	E103°56′26.5″，N25°53′39.4″	Scattered distribution	*R. irroratum*、*R. decorum*
A	Ailao Mountains	Jingdong, Pu’er	2483 m	E101°1′15.6″，N24°31′15.6″	Concentrated distribution	*R.yunnanense*
C	Huadianba, Cangshan Mountain	Dali	2601 m	E100°2′17.2″，N25°53′12.1″	Concentrated distribution	*R. yunnanense*

Table S2. Soil physicochemical properties of the four *Rhododendron yunnanense* sampling sites. Values are presented as mean ± SD (n = 3 per sampling site).

Sampling site（Elevation）	Junzi Mountain （2252m）	Source of the Pearl River （2305m）	Ailao Mountains （2483m）	Huadianba, Cangshan Mountain （2601m）
SOM(g/kg)	375.15±175.46	497.12±141.11	314.74±55.25	78.9±37.62
TN(g/kg)	9.17±4.11	13.42±1.51	8.84±0.74	2.64±0.25
TP(g/kg)	1.61±0.61	0.75±0.12	0.91±0.11	1.02±0.23
TK(g/kg)	6.02±2.79	4.94±1.90	11.50±1.32	13.88±2.04
HN(mg/kg)	484.63±157.38	645.87±186.82	544.00±46.66	205.60±32.40
AP(mg/kg)	75.95±66.32	57.67±20.86	47.39±24.83	21.82±6.36
AK(mg/kg)	368.10±78.37	386.87±119.51	178.55±9.29	123.8±5.45
pH	5.21±0.80	4.09±0.41	4.63±0.48	5.43±0.38

Table S3. Stable ASV sharing and overlap between the rhizosphere and endosphere across four sampling sites

Microbial group	Sampling site	Rhizosphere stable ASVs	Endosphere stable ASVs	Shared stable ASVs	Rhizosphere-specific ASVs	Endosphere-specific ASVs	Rhizosphere-referenced overlap (%)
Bacteria	JZ	39	311	8	31	303	20.5
Bacteria	ZJY	46	392	19	27	373	41.3
Bacteria	AL	64	842	23	41	819	35.9
Bacteria	CS	73	348	13	60	335	17.8
Fungi	JZ	12	34	3	9	31	25.0
Fungi	ZJY	19	50	6	13	44	31.6
Fungi	AL	28	38	1	27	37	3.6
Fungi	CS	15	103	8	7	95	53.3

Table S4. Overall core ASV overlap between rhizosphere and endosphere bacterial and fungal communities

Microbial group	Rhizosphere core ASVs	Endosphere core ASVs	Shared core ASVs	Rhizosphere-specific core ASVs	Endosphere-specific core ASVs
Bacteria	6	33	3	3	30
Fungi	1	3	0	1	3

Table S5. Site-specific core ASV overlap and Jaccard similarity between rhizosphere and endosphere communities

Microbial group	Sampling site	Rhizosphere core ASVs	Endosphere core ASVs	Shared core ASVs	Rhizosphere-specific core ASVs	Endosphere-specific core ASVs	Core union	Jaccard similarity (%)
Bacteria	JZ	37	299	8	29	291	328	2.4
Bacteria	ZJY	46	373	18	28	355	401	4.5
Bacteria	AL	62	776	22	40	754	816	2.7
Bacteria	CS	73	345	13	60	332	405	3.2
Fungi	JZ	10	34	3	7	31	41	7.3
Fungi	ZJY	16	47	4	12	43	59	6.8
Fungi	AL	27	35	1	26	34	61	1.6
Fungi	CS	12	84	4	8	80	92	4.3

Table S6. Complete genus-level compartment association results from indicator and paired abundance analyses

**Microbial group**	**Genus**	**Phylum**	**Family**	**Indicator group**	**IndVal.g**	**IndVal raw P**	**IndVal FDR**	**IndVal significant**	**Paired abundance direction**
Bacteria	0319-6G20	Bdellovibrionota	0319-6G20	Endosphere	0.8623	0.0190	0.0454	Yes	Endosphere

Microbial group	Genus	Phylum	Family	Indicator group	IndVal.g	IndVal raw P	IndVal FDR	IndVal significant	Paired abundance direction
Bacteria	0319-7L14	Actinobacteriota	0319-7L14	Endosphere	0.6455	0.0650	0.1151	No	Endosphere
Bacteria	37-13	Bacteroidota	37-13	Endosphere	0.7071	0.0240	0.0538	No	Endosphere
Bacteria	67-14	Actinobacteriota	67-14	Rhizosphere	0.3759	0.8870	0.9874	No	Rhizosphere
Bacteria	A21b	Proteobacteria	A21b	Endosphere	0.9574	0.0020	0.0098	Yes	Endosphere
Bacteria	AAP99	Proteobacteria	Sutterellaceae	Rhizosphere	0.3600	0.7530	0.8724	No	Rhizosphere
Bacteria	Acidibacter	Proteobacteria	Unknown_Family	Endosphere	0.7002	0.6620	0.7812	No	Endosphere
Bacteria	Acidicaldus	Proteobacteria	Acetobacteraceae	Endosphere	0.9056	0.0010	0.0077	Yes	Endosphere
Bacteria	Acidicapsa	Acidobacteriota	Acidobacteriaceae_(Subgroup_1)	Endosphere	0.5026	0.4500	0.5570	No	Endosphere
Bacteria	Acidiphilium	Proteobacteria	Acetobacteraceae	Endosphere	0.6274	0.0550	0.1025	No	Endosphere
Bacteria	Acidipila-Silvibacterium	Acidobacteriota	Acidobacteriaceae_(Subgroup_1)	Endosphere	0.8136	0.0040	0.0157	Yes	Endosphere
Bacteria	Acidisoma	Proteobacteria	Acetobacteraceae	Endosphere	0.8840	0.0060	0.0190	Yes	Endosphere
Bacteria	Acidocella	Proteobacteria	Acetobacteraceae	Rhizosphere	0.5177	0.9980	1.0000	No	Rhizosphere

Microbial group	Genus	Phylum	Family	Indicator group	IndVal.g	IndVal raw P	IndVal FDR	IndVal significant	Paired abundance direction
Bacteria	Acidothermus	Actinobacteriota	Acidothermaceae	Rhizosphere	0.9176	0.0040	0.0157	Yes	Rhizosphere
Bacteria	Actinoallomurus	Actinobacteriota	Thermomonosporaceae	Endosphere	0.8030	0.0120	0.0312	Yes	Endosphere
Bacteria	Actinospica	Actinobacteriota	Actinospicaceae	Rhizosphere	0.5543	0.4350	0.5422	No	Rhizosphere
Bacteria	Afipia	Proteobacteria	Xanthobacteraceae	Rhizosphere	0.4442	0.9060	1.0000	No	Rhizosphere
Bacteria	Aliidongia	Proteobacteria	Elsteraceae	Rhizosphere	0.3838	0.5250	0.6279	No	Rhizosphere
Bacteria	Allorhizobium-Neorhizobium-Pararhizobium-Rhizobium	Proteobacteria	Rhizobiaceae	Rhizosphere	0.7676	0.1870	0.2648	No	Rhizosphere
Bacteria	Altererythrobacter	Proteobacteria	Sphingomonadaceae	Endosphere	0.4007	0.4790	0.5847	No	Endosphere
Bacteria	Amb-16S-1323	Proteobacteria	Amb-16S-1323	Endosphere	0.7071	0.0300	0.0640	No	Endosphere
Bacteria	Anaeromyxobacter	Myxococcota	Anaeromyxobacteraceae	Endosphere	1.0000	0.0010	0.0077	Yes	Endosphere
Bacteria	Asticcacaulis	Proteobacteria	Caulobacteraceae	Endosphere	0.5914	0.0530	0.0998	No	Endosphere
Bacteria	B1-7BS	Proteobacteria	B1-7BS	Endosphere	0.8165	0.0080	0.0232	Yes	Endosphere
Bacteria	Bacillus	Firmicutes	Bacillaceae	Rhizosphere	0.6133	0.9740	1.0000	No	Rhizosphere

Microbial group	Genus	Phylum	Family	Indicator group	IndVal.g	IndVal raw P	IndVal FDR	IndVal significant	Paired abundance direction
Bacteria	bacteriap25	Myxococcota	bacteriap25	Endosphere	0.5000	0.2620	0.3461	No	Endosphere
Bacteria	Bauldia	Proteobacteria	Rhizobiales_Incertae_Sedis	Endosphere	0.9574	0.0020	0.0098	Yes	Endosphere
Bacteria	Bdellovibrio	Bdellovibrionota	Bdellovibrionaceae	Endosphere	0.9891	0.0010	0.0077	Yes	Endosphere
Bacteria	BIrii41	Myxococcota	BIrii41	Endosphere	0.8660	0.0030	0.0133	Yes	Endosphere
Bacteria	Blfdi19	Myxococcota	Blfdi19	Endosphere	0.9041	0.0030	0.0133	Yes	Endosphere
Bacteria	Bradyrhizobium	Proteobacteria	Xanthobacteraceae	Rhizosphere	0.7936	0.3720	0.4703	No	Rhizosphere
Bacteria	Brevundimonas	Proteobacteria	Caulobacteraceae	Rhizosphere	0.5379	0.9870	1.0000	No	Rhizosphere
Bacteria	BSV26	Bacteroidota	BSV26	Endosphere	0.6455	0.0600	0.1095	No	Endosphere
Bacteria	Burkholderia-Caballeronia-Paraburkholderia	Proteobacteria	Burkholderiaceae	Rhizosphere	0.6775	0.9700	1.0000	No	Rhizosphere
Bacteria	Candidatus_Berkiella	Proteobacteria	Unknown_Family	Endosphere	0.9574	0.0010	0.0077	Yes	Endosphere
Bacteria	Candidatus_Ovatusbacter	Proteobacteria	Unknown_Family	Endosphere	0.8165	0.0040	0.0157	Yes	Endosphere
Bacteria	Caulobacter	Proteobacteria	Caulobacteraceae	Endosphere	0.4685	0.8810	0.9874	No	Rhizosphere

Microbial group	Genus	Phylum	Family	Indicator group	IndVal.g	IndVal raw P	IndVal FDR	IndVal significant	Paired abundance direction
Bacteria	CL500-29_marine_group	Actinobacteriota	Ilumatobacteraceae	Endosphere	0.5774	0.1080	0.1754	No	Endosphere
Bacteria	Collimonas	Proteobacteria	Oxalobacteraceae	Endosphere	0.2866	1.0000	1.0000	No	Rhizosphere
Bacteria	Comamonas	Proteobacteria	Comamonadaceae	Endosphere	0.3300	0.7590	0.8724	No	Endosphere
Bacteria	Conexibacter	Actinobacteriota	Solirubrobacteraceae	Endosphere	0.9602	0.0010	0.0077	Yes	Endosphere
Bacteria	Cupriavidus	Proteobacteria	Burkholderiaceae	Rhizosphere	0.3387	0.9470	1.0000	No	Rhizosphere
Bacteria	Devosia	Proteobacteria	Devosiaceae	Endosphere	0.3888	0.6080	0.7223	No	Endosphere
Bacteria	Dokdonella	Proteobacteria	Rhodanobacteraceae	Endosphere	0.7071	0.0310	0.0646	No	Endosphere
Bacteria	Dongia	Proteobacteria	Dongiaceae	Endosphere	0.7259	0.0290	0.0626	No	Endosphere
Bacteria	Duganella	Proteobacteria	Oxalobacteraceae	Rhizosphere	0.4078	0.9330	1.0000	No	Endosphere
Bacteria	Dyella	Proteobacteria	Rhodanobacteraceae	Endosphere	0.7144	0.0370	0.0736	No	Endosphere
Bacteria	Edaphobacter	Acidobacteriota	Acidobacteriaceae_(Subgroup_1)	Endosphere	0.9129	0.0010	0.0077	Yes	Endosphere
Bacteria	Ellin6055	Proteobacteria	Sphingomonadaceae	Endosphere	0.5699	0.1670	0.2403	No	Endosphere

Microbial group	Genus	Phylum	Family	Indicator group	IndVal.g	IndVal raw P	IndVal FDR	IndVal significant	Paired abundance direction
Bacteria	Ellin6067	Proteobacteria	Nitrosomonadaceae	Endosphere	0.9451	0.0020	0.0098	Yes	Endosphere
Bacteria	Erwinia	Proteobacteria	Erwiniaceae	Rhizosphere	0.6874	0.0900	0.1517	No	Rhizosphere
Bacteria	FCPU426	FCPU426	FCPU426	Endosphere	0.7638	0.0190	0.0454	Yes	Endosphere
Bacteria	Ferritrophicum	Proteobacteria	Sulfuricellaceae	Endosphere	0.5774	0.1330	0.1995	No	Endosphere
Bacteria	Gaiella	Actinobacteriota	Gaiellaceae	Endosphere	0.5774	0.1160	0.1850	No	Endosphere
Bacteria	Gemmatimonas	Gemmatimonadota	Gemmatimonadaceae	Endosphere	0.8811	0.0080	0.0232	Yes	Endosphere
Bacteria	GOUTA6	Proteobacteria	Nitrosomonadaceae	Endosphere	0.7071	0.0340	0.0692	No	Endosphere
Bacteria	Granulicella	Acidobacteriota	Acidobacteriaceae_(Subgroup_1)	Endosphere	0.9333	0.0010	0.0077	Yes	Endosphere
Bacteria	Haliangium	Myxococcota	Haliangiaceae	Endosphere	0.9250	0.0030	0.0133	Yes	Endosphere
Bacteria	Herbaspirillum	Proteobacteria	Oxalobacteraceae	Rhizosphere	0.3383	0.9260	1.0000	No	Endosphere
Bacteria	Hirschia	Proteobacteria	Hyphomonadaceae	Endosphere	0.9129	0.0030	0.0133	Yes	Endosphere
Bacteria	Hyphomicrobium	Proteobacteria	Hyphomicrobiaceae	Endosphere	0.4859	0.5200	0.6261	No	Rhizosphere

Microbial group	Genus	Phylum	Family	Indicator group	IndVal.g	IndVal raw P	IndVal FDR	IndVal significant	Paired abundance direction
Bacteria	Iamia	Actinobacteriota	Iamiaceae	Endosphere	0.8468	0.0180	0.0449	Yes	Endosphere
Bacteria	IMCC26256	Actinobacteriota	IMCC26256	Endosphere	0.9937	0.0010	0.0077	Yes	Endosphere
Bacteria	Inquilinus	Proteobacteria	Inquilinaceae	Endosphere	0.7246	0.1360	0.2004	No	Endosphere
Bacteria	IS-44	Proteobacteria	Nitrosomonadaceae	Endosphere	0.7071	0.0310	0.0646	No	Endosphere
Bacteria	JG36-TzT-191	Proteobacteria	JG36-TzT-191	Endosphere	0.8165	0.0050	0.0174	Yes	Endosphere
Bacteria	Kapabacteriales	Bacteroidota	Kapabacteriales	Endosphere	0.6455	0.0580	0.1069	No	Endosphere
Bacteria	KF-JG30-C25	Proteobacteria	KF-JG30-C25	Endosphere	0.8528	0.0040	0.0157	Yes	Endosphere
Bacteria	Kitasatospora	Actinobacteriota	Streptomycetaceae	Endosphere	0.7071	0.0320	0.0659	No	Endosphere
Bacteria	Labrys	Proteobacteria	Labraceae	Rhizosphere	0.4348	0.8860	0.9874	No	Rhizosphere
Bacteria	Legionella	Proteobacteria	Legionellaceae	Rhizosphere	0.2877	1.0000	1.0000	No	Rhizosphere
Bacteria	Limosilactobacillus	Firmicutes	Lactobacillaceae	Rhizosphere	0.5000	0.2420	0.3234	No	Rhizosphere
Bacteria	Massilia	Proteobacteria	Oxalobacteraceae	Rhizosphere	0.6993	0.7850	0.8964	No	Rhizosphere

Microbial group	Genus	Phylum	Family	Indicator group	IndVal.g	IndVal raw P	IndVal FDR	IndVal significant	Paired abundance direction
Bacteria	MBNT15	MBNT15	MBNT15	Endosphere	0.8165	0.0100	0.0272	Yes	Endosphere
Bacteria	Mesorhizobium	Proteobacteria	Rhizobiaceae	Endosphere	0.7867	0.0430	0.0818	No	Endosphere
Bacteria	Methylobacterium-Methylorubrum	Proteobacteria	Beijerinckiaceae	Rhizosphere	0.7855	0.5110	0.6195	No	Rhizosphere
Bacteria	Methyloligellaceae	Proteobacteria	Methyloligellaceae	Endosphere	0.5774	0.1480	0.2147	No	Endosphere
Bacteria	mle1-7	Proteobacteria	Nitrosomonadaceae	Endosphere	0.6455	0.0800	0.1362	No	Endosphere
Bacteria	MND1	Proteobacteria	Nitrosomonadaceae	Endosphere	0.9129	0.0020	0.0098	Yes	Endosphere
Bacteria	MSB-4B10	Myxococcota	MSB-4B10	Endosphere	0.9129	0.0020	0.0098	Yes	Endosphere
Bacteria	Myxococcaceae	Myxococcota	Myxococcaceae	Endosphere	0.8165	0.0100	0.0272	Yes	Endosphere
Bacteria	Nitrospira	Nitrospirota	Nitrospiraceae	Endosphere	0.8165	0.0080	0.0232	Yes	Endosphere
Bacteria	Novosphingobium	Proteobacteria	Sphingomonadaceae	Rhizosphere	0.6209	0.4310	0.5410	No	Rhizosphere
Bacteria	Occallatibacter	Acidobacteriota	Acidobacteriaceae_(Subgroup_1)	Endosphere	0.9052	0.0020	0.0098	Yes	Endosphere
Bacteria	Ochrobactrum	Proteobacteria	Rhizobiaceae	Rhizosphere	0.5000	0.2740	0.3566	No	Rhizosphere

Microbial group	Genus	Phylum	Family	Indicator group	IndVal.g	IndVal raw P	IndVal FDR	IndVal significant	Paired abundance direction
Bacteria	OM190	Planctomycetota	OM190	Endosphere	0.5774	0.1310	0.1982	No	Endosphere
Bacteria	OM27_clade	Bdellovibrionota	Bdellovibrionaceae	Endosphere	0.5000	0.2410	0.3234	No	Endosphere
Bacteria	OM60(NOR5)_clade	Proteobacteria	Halieaceae	Endosphere	0.5000	0.2430	0.3234	No	Endosphere
Bacteria	P3OB-42	Myxococcota	Myxococcaceae	Endosphere	0.7334	0.0220	0.0506	No	Endosphere
Bacteria	Paenibacillus	Firmicutes	Paenibacillaceae	Rhizosphere	0.4927	0.9610	1.0000	No	Rhizosphere
Bacteria	Pajaroellobacter	Myxococcota	Polyangiaceae	Endosphere	0.7953	0.0170	0.0436	Yes	Endosphere
Bacteria	Pandoraea	Proteobacteria	Burkholderiaceae	Rhizosphere	0.3905	0.7560	0.8724	No	Rhizosphere
Bacteria	PB19	Desulfobacterota	PB19	Endosphere	0.5774	0.1100	0.1770	No	Endosphere
Bacteria	Pedomicrobium	Proteobacteria	Hyphomicrobiaceae	Endosphere	0.7532	0.0350	0.0704	No	Endosphere
Bacteria	Phenylobacterium	Proteobacteria	Caulobacteraceae	Endosphere	0.8804	0.0050	0.0174	Yes	Endosphere
Bacteria	Plot4-2H12	Proteobacteria	Sphingomonadaceae	Endosphere	0.9416	0.0020	0.0098	Yes	Endosphere
Bacteria	Pseudolabrys	Proteobacteria	Xanthobacteraceae	Endosphere	0.8217	0.0050	0.0174	Yes	Endosphere

Microbial group	Genus	Phylum	Family	Indicator group	IndVal.g	IndVal raw P	IndVal FDR	IndVal significant	Paired abundance direction
Bacteria	Pseudomonas	Proteobacteria	Pseudomonadaceae	Rhizosphere	0.9919	0.0060	0.0190	Yes	Rhizosphere
Bacteria	Pseudonocardia	Actinobacteriota	Pseudonocardiaceae	Endosphere	0.7964	0.0220	0.0506	No	Endosphere
Bacteria	Pseudorhodoplanes	Proteobacteria	Xanthobacteraceae	Endosphere	0.5000	0.2210	0.3070	No	Endosphere
Bacteria	RCP2-54	RCP2-54	RCP2-54	Endosphere	0.9908	0.0010	0.0077	Yes	Endosphere
Bacteria	Reyranella	Proteobacteria	Reyranellaceae	Endosphere	0.9459	0.0020	0.0098	Yes	Endosphere
Bacteria	Rhizobacter	Proteobacteria	Comamonadaceae	Endosphere	0.4726	0.8750	0.9874	No	Rhizosphere
Bacteria	Rhodanobacter	Proteobacteria	Rhodanobacteraceae	Endosphere	0.8627	0.0050	0.0174	Yes	Endosphere
Bacteria	Rhodomicrobium	Proteobacteria	Rhodomicrobiaceae	Endosphere	0.4303	1.0000	1.0000	No	Rhizosphere
Bacteria	Rhodoplanes	Proteobacteria	Xanthobacteraceae	Endosphere	0.7071	0.0430	0.0818	No	Endosphere
Bacteria	Rhodopseudomonas	Proteobacteria	Xanthobacteraceae	Rhizosphere	0.4721	0.4730	0.5814	No	Rhizosphere
Bacteria	Rhodovastum	Proteobacteria	Acetobacteraceae	Endosphere	0.8616	0.0050	0.0174	Yes	Endosphere
Bacteria	Roseiarcus	Proteobacteria	Beijerinckiaceae	Endosphere	0.9610	0.0010	0.0077	Yes	Endosphere

Microbial group	Genus	Phylum	Family	Indicator group	IndVal.g	IndVal raw P	IndVal FDR	IndVal significant	Paired abundance direction
Bacteria	Roseomonas	Proteobacteria	Acetobacteraceae	Rhizosphere	0.2567	1.0000	1.0000	No	Rhizosphere
Bacteria	Sandaracinus	Myxococcota	Sandaracinaceae	Endosphere	0.3234	1.0000	1.0000	No	Rhizosphere
Bacteria	SAR324_clade(Marine_group_B)	SAR324_clade(Marine_group_B)	SAR324_clade(Marine_group_B)	Endosphere	0.6455	0.0700	0.1227	No	Endosphere
Bacteria	SC-I-84	Proteobacteria	SC-I-84	Endosphere	0.9574	0.0020	0.0098	Yes	Endosphere
Bacteria	Serratia	Proteobacteria	Yersiniaceae	Rhizosphere	0.5772	0.1360	0.2004	No	Rhizosphere
Bacteria	Shewanella	Proteobacteria	Shewanellaceae	Rhizosphere	0.5000	0.2760	0.3566	No	Rhizosphere
Bacteria	SJA-28	Bacteroidota	SJA-28	Endosphere	0.5774	0.1080	0.1754	No	Endosphere
Bacteria	SM2D12	Proteobacteria	SM2D12	Endosphere	0.9887	0.0010	0.0077	Yes	Endosphere
Bacteria	Sphingobium	Proteobacteria	Sphingomonadaceae	Rhizosphere	0.3697	0.9340	1.0000	No	Rhizosphere
Bacteria	Sphingomonas	Proteobacteria	Sphingomonadaceae	Rhizosphere	0.8411	0.9980	1.0000	No	Rhizosphere
Bacteria	Staphylococcus	Firmicutes	Staphylococcaceae	Rhizosphere	0.5774	0.1260	0.1938	No	Rhizosphere
Bacteria	Streptacidiphilus	Actinobacteriota	Streptomycetaceae	Endosphere	0.5406	0.1760	0.2512	No	Endosphere

Microbial group	Genus	Phylum	Family	Indicator group	IndVal.g	IndVal raw P	IndVal FDR	IndVal significant	Paired abundance direction
Bacteria	Streptomyces	Actinobacteriota	Streptomycetaceae	Rhizosphere	0.3378	1.0000	1.0000	No	Rhizosphere
Bacteria	Subgroup_10	Acidobacteriota	Thermoanaerobaculaceae	Endosphere	0.8660	0.0060	0.0190	Yes	Endosphere
Bacteria	Subgroup_11	Acidobacteriota	Subgroup_11	Endosphere	0.5000	0.2330	0.3172	No	Endosphere
Bacteria	Subgroup_13	Acidobacteriota	Subgroup_13	Endosphere	0.5774	0.1250	0.1938	No	Endosphere
Bacteria	Subgroup_15	Acidobacteriota	Subgroup_15	Endosphere	0.7638	0.0120	0.0312	Yes	Endosphere
Bacteria	Subgroup_22	Acidobacteriota	Subgroup_22	Endosphere	0.5774	0.1180	0.1865	No	Endosphere
Bacteria	Subgroup_5	Acidobacteriota	Subgroup_5	Endosphere	0.6455	0.0760	0.1306	No	Endosphere
Bacteria	Subgroup_7	Acidobacteriota	Subgroup_7	Endosphere	0.7627	0.0190	0.0454	Yes	Endosphere
Bacteria	SWB02	Proteobacteria	Hyphomonadaceae	Endosphere	0.8113	0.0080	0.0232	Yes	Endosphere
Bacteria	TRA3-20	Proteobacteria	TRA3-20	Endosphere	0.8660	0.0050	0.0174	Yes	Endosphere
Bacteria	unclassified_Acetobacteraceae	Proteobacteria	Acetobacteraceae	Endosphere	0.9853	0.0010	0.0077	Yes	Endosphere
Bacteria	unclassified_Acidimicrobiia	Actinobacteriota	unclassified_Acidimicrobiia	Endosphere	0.9961	0.0010	0.0077	Yes	Endosphere

Microbial group	Genus	Phylum	Family	Indicator group	IndVal.g	IndVal raw P	IndVal FDR	IndVal significant	Paired abundance direction
Bacteria	unclassified_Acidobacteriaceae_(Subgroup_1)	Acidobacteriota	Acidobacteriaceae_(Subgroup_1)	Endosphere	0.9455	0.0010	0.0077	Yes	Endosphere
Bacteria	unclassified_Acidobacteriales	Acidobacteriota	unclassified_Acidobacteriales	Endosphere	1.0000	0.0010	0.0077	Yes	Endosphere
Bacteria	unclassified_Acidobacteriota	Acidobacteriota	unclassified_Acidobacteriota	Endosphere	0.9574	0.0020	0.0098	Yes	Endosphere
Bacteria	unclassified_Actinobacteria	Actinobacteriota	unclassified_Actinobacteria	Endosphere	0.7363	0.2130	0.2992	No	Endosphere
Bacteria	unclassified_Actinobacteriota	Actinobacteriota	unclassified_Actinobacteriota	Endosphere	0.9824	0.0010	0.0077	Yes	Endosphere
Bacteria	unclassified_Alcaligenaceae	Proteobacteria	Alcaligenaceae	Rhizosphere	0.5690	0.1370	0.2004	No	Rhizosphere
Bacteria	unclassified_Alphaproteobacteria	Proteobacteria	unclassified_Alphaproteobacteria	Endosphere	0.9482	0.0010	0.0077	Yes	Endosphere
Bacteria	unclassified_Bacteria	unclassified_Bacteria	unclassified_Bacteria	Endosphere	0.9762	0.0010	0.0077	Yes	Endosphere
Bacteria	unclassified_Beijerinckiaceae	Proteobacteria	Beijerinckiaceae	Endosphere	0.9510	0.0010	0.0077	Yes	Endosphere
Bacteria	unclassified_Burkholderiaceae	Proteobacteria	Burkholderiaceae	Endosphere	0.5200	0.2220	0.3070	No	Endosphere
Bacteria	unclassified_Burkholderiales	Proteobacteria	unclassified_Burkholderiales	Endosphere	0.9041	0.0060	0.0190	Yes	Endosphere
Bacteria	unclassified_Caulobacteraceae	Proteobacteria	Caulobacteraceae	Endosphere	0.9367	0.0010	0.0077	Yes	Endosphere

Microbial group	Genus	Phylum	Family	Indicator group	IndVal.g	IndVal raw P	IndVal FDR	IndVal significant	Paired abundance direction
Bacteria	unclassified_Comamonadaceae	Proteobacteria	Comamonadaceae	Endosphere	0.7225	0.0620	0.1120	No	Endosphere
Bacteria	unclassified_Elsterales	Proteobacteria	unclassified_Elsterales	Endosphere	0.9574	0.0010	0.0077	Yes	Endosphere
Bacteria	unclassified_Enterobacterales	Proteobacteria	unclassified_Enterobacterales	Rhizosphere	0.7048	0.0760	0.1306	No	Rhizosphere
Bacteria	unclassified_Enterobacteriaceae	Proteobacteria	Enterobacteriaceae	Rhizosphere	0.8079	0.0410	0.0797	No	Rhizosphere
Bacteria	unclassified_Frankiales	Actinobacteriota	unclassified_Frankiales	Endosphere	0.6916	0.1000	0.1654	No	Endosphere
Bacteria	unclassified_Gaiellales	Actinobacteriota	unclassified_Gaiellales	Endosphere	0.9574	0.0020	0.0098	Yes	Endosphere
Bacteria	unclassified_Gammaproteobacteria	Proteobacteria	unclassified_Gammaproteobacteria	Endosphere	0.8313	0.0040	0.0157	Yes	Endosphere
Bacteria	unclassified_Gemmatimonadaceae	Gemmatimonadota	Gemmatimonadaceae	Endosphere	0.7071	0.0280	0.0612	No	Endosphere
Bacteria	unclassified_Hyphomicrobiaceae	Proteobacteria	Hyphomicrobiaceae	Endosphere	0.8165	0.0100	0.0272	Yes	Endosphere
Bacteria	unclassified_Methylophilaceae	Proteobacteria	Methylophilaceae	Endosphere	0.5774	0.1220	0.1911	No	Endosphere
Bacteria	unclassified_Microbacteriaceae	Actinobacteriota	Microbacteriaceae	Endosphere	0.4568	0.3550	0.4521	No	Endosphere
Bacteria	unclassified_Microtrichales	Actinobacteriota	unclassified_Microtrichales	Endosphere	0.7058	0.0250	0.0553	No	Endosphere

Microbial group	Genus	Phylum	Family	Indicator group	IndVal.g	IndVal raw P	IndVal FDR	IndVal significant	Paired abundance direction
Bacteria	unclassified_Myxococcaceae	Myxococcota	Myxococcaceae	Endosphere	0.9870	0.0010	0.0077	Yes	Endosphere
Bacteria	unclassified_Myxococcales	Myxococcota	unclassified_Myxococcales	Endosphere	0.6049	0.1270	0.1938	No	Endosphere
Bacteria	unclassified_Oxalobacteraceae	Proteobacteria	Oxalobacteraceae	Rhizosphere	0.7973	0.2660	0.3488	No	Rhizosphere
Bacteria	unclassified_Proteobacteria	Proteobacteria	unclassified_Proteobacteria	Endosphere	0.8206	0.0220	0.0506	No	Endosphere
Bacteria	unclassified_Rhizobiaceae	Proteobacteria	Rhizobiaceae	Rhizosphere	0.5084	0.3110	0.3989	No	Rhizosphere
Bacteria	unclassified_Rhizobiales	Proteobacteria	unclassified_Rhizobiales	Endosphere	0.9492	0.0100	0.0272	Yes	Endosphere
Bacteria	unclassified_Rhodanobacteraceae	Proteobacteria	Rhodanobacteraceae	Endosphere	0.9359	0.0010	0.0077	Yes	Endosphere
Bacteria	unclassified_Solirubrobacteraceae	Actinobacteriota	Solirubrobacteraceae	Endosphere	0.9388	0.0020	0.0098	Yes	Endosphere
Bacteria	unclassified_Solirubrobacterales	Actinobacteriota	unclassified_Solirubrobacterales	Endosphere	0.8484	0.0120	0.0312	Yes	Endosphere
Bacteria	unclassified_Sphingomonadaceae	Proteobacteria	Sphingomonadaceae	Endosphere	0.4994	0.6730	0.7889	No	Endosphere
Bacteria	unclassified_Streptosporangiales	Actinobacteriota	unclassified_Streptosporangiales	Endosphere	0.8605	0.0020	0.0098	Yes	Endosphere
Bacteria	unclassified_Thermomonosporaceae	Actinobacteriota	Thermomonosporaceae	Endosphere	0.7500	0.0240	0.0538	No	Endosphere

Microbial group	Genus	Phylum	Family	Indicator group	IndVal.g	IndVal raw P	IndVal FDR	IndVal significant	Paired abundance direction
Bacteria	unclassified_Xanthobacteraceae	Proteobacteria	Xanthobacteraceae	Rhizosphere	0.7819	0.9620	1.0000	No	Rhizosphere
Bacteria	unclassified_Xanthomonadaceae	Proteobacteria	Xanthomonadaceae	Endosphere	0.8165	0.0060	0.0190	Yes	Endosphere
Bacteria	URHD0088	Proteobacteria	URHD0088	Endosphere	0.6294	0.1000	0.1654	No	Endosphere
Bacteria	vadinHA49	Planctomycetota	vadinHA49	Endosphere	0.7374	0.0390	0.0767	No	Endosphere
Bacteria	Variovorax	Proteobacteria	Comamonadaceae	Endosphere	0.7638	0.0180	0.0449	Yes	Endosphere
Bacteria	Vicinamibacteraceae	Acidobacteriota	Vicinamibacteraceae	Endosphere	0.6455	0.0640	0.1144	No	Endosphere
Bacteria	WD260	Proteobacteria	WD260	Endosphere	0.8165	0.0070	0.0217	Yes	Endosphere
Bacteria	Xanthobacteraceae	Proteobacteria	Xanthobacteraceae	Endosphere	0.7103	0.2260	0.3101	No	Endosphere
Fungi	Acarospora	Ascomycota	Acarosporaceae	Endosphere	0.4996	0.3640	0.5123	No	Endosphere
Fungi	Acremonium	Ascomycota	Hypocreales_fam_Incertae_sedis	Endosphere	0.5774	0.1210	0.3342	No	Endosphere
Fungi	Agaricomycetes_gen_Incertae_sedis	Basidiomycota	Agaricomycetes_fam_Incertae_sedis	Endosphere	0.5000	0.2560	0.3998	No	Endosphere
Fungi	Albophoma	Ascomycota	Ophiocordycipitaceae	Endosphere	0.7636	0.0220	0.1045	No	Endosphere

Microbial group	Genus	Phylum	Family	Indicator group	IndVal.g	IndVal raw P	IndVal FDR	IndVal significant	Paired abundance direction
Fungi	Alfoldia	Ascomycota	Amorosiaceae	Endosphere	0.3521	0.5030	0.6240	No	Endosphere
Fungi	Amanita	Basidiomycota	Amanitaceae	Endosphere	0.4076	0.4990	0.6240	No	Endosphere
Fungi	Amaurodon	Basidiomycota	Thelephoraceae	Endosphere	0.5774	0.1180	0.3342	No	Endosphere
Fungi	Amplistromataceae_gen_Incertae_sedis	Ascomycota	Amplistromataceae	Endosphere	0.5774	0.1500	0.3563	No	Endosphere
Fungi	Apiotrichum	Basidiomycota	Trichosporonaceae	Endosphere	0.5774	0.1260	0.3342	No	Endosphere
Fungi	Archaeorhizomyces	Ascomycota	Archaeorhizomycetaceae	Endosphere	0.8953	0.0100	0.0760	No	Endosphere
Fungi	Arcopilus	Ascomycota	Chaetomiaceae	Endosphere	0.5774	0.1140	0.3342	No	Endosphere
Fungi	Aspergillus	Ascomycota	Aspergillaceae	Endosphere	0.5000	0.2490	0.3998	No	Endosphere
Fungi	Atractospora	Ascomycota	Myrmecridiales_fam_Incertae_sedis	Rhizosphere	0.2854	1.0000	1.0000	No	Rhizosphere
Fungi	Beauveria	Ascomycota	Cordycipitaceae	Endosphere	0.7071	0.0380	0.1547	No	Endosphere
Fungi	Botryobasidium	Basidiomycota	Botryobasidiaceae	Endosphere	0.5000	0.2630	0.3998	No	Endosphere
Fungi	Capnodiaceae_gen_Incertae_sedis	Ascomycota	Capnodiaceae	Endosphere	0.5000	0.2470	0.3998	No	Endosphere

Microbial group	Genus	Phylum	Family	Indicator group	IndVal.g	IndVal raw P	IndVal FDR	IndVal significant	Paired abundance direction
Fungi	Capnodiales_gen_Incertae_sedis	Ascomycota	Capnodiales_fam_Incertae_sedis	Endosphere	0.5734	0.2080	0.3998	No	Endosphere
Fungi	Capronia	Ascomycota	Herpotrichiellaceae	Endosphere	0.6393	0.0700	0.2414	No	Endosphere
Fungi	Cenococcum	Ascomycota	Gloniaceae	Endosphere	0.4998	0.2470	0.3998	No	Endosphere
Fungi	Cephalotheca	Ascomycota	Cephalothecaceae	Rhizosphere	0.4165	0.8910	0.9493	No	Rhizosphere
Fungi	Ceratobasidiaceae_gen_Incertae_sedis	Basidiomycota	Ceratobasidiaceae	Endosphere	0.4586	0.4520	0.6062	No	Endosphere
Fungi	Cercospora	Ascomycota	Mycosphaerellaceae	Endosphere	0.5131	0.4040	0.5483	No	Endosphere
Fungi	Chaetosphaeria	Ascomycota	Chaetosphaeriaceae	Endosphere	0.6086	0.3620	0.5123	No	Endosphere
Fungi	Chaetothyriales_gen_Incertae_sedis	Ascomycota	Chaetothyriales_fam_Incertae_sedis	Endosphere	0.3856	1.0000	1.0000	No	Rhizosphere
Fungi	Cistella	Ascomycota	Hyaloscyphaceae	Rhizosphere	0.5127	0.5620	0.6605	No	Rhizosphere
Fungi	Cladophialophora	Ascomycota	Herpotrichiellaceae	Rhizosphere	0.6456	0.6570	0.7490	No	Rhizosphere
Fungi	Cladosporium	Ascomycota	Cladosporiaceae	Endosphere	0.7043	0.1680	0.3909	No	Endosphere
Fungi	Clavaria	Basidiomycota	Clavariaceae	Endosphere	0.5774	0.1430	0.3469	No	Endosphere

Microbial group	Genus	Phylum	Family	Indicator group	IndVal.g	IndVal raw P	IndVal FDR	IndVal significant	Paired abundance direction
Fungi	Coniochaeta	Ascomycota	Coniochaetaceae	Endosphere	0.5774	0.1290	0.3342	No	Endosphere
Fungi	Exophiala	Ascomycota	Herpotrichiellaceae	Endosphere	0.7638	0.0170	0.0969	No	Endosphere
Fungi	Fungi_gen_Incertae_sedis	Fungi_phy_Incertae_sedis	Fungi_fam_Incertae_sedis	Endosphere	0.9996	0.0010	0.0127	Yes	Endosphere
Fungi	Fusarium	Ascomycota	Nectriaceae	Endosphere	0.3516	0.9700	1.0000	No	Endosphere
Fungi	Geminibasidium	Basidiomycota	Geminibasidiaceae	Endosphere	0.7634	0.0140	0.0939	No	Endosphere
Fungi	Glutinomyces	Ascomycota	Hyaloscyphaceae	Rhizosphere	0.3864	0.5200	0.6240	No	Rhizosphere
Fungi	GS11_gen_Incertae_sedis	Rozellomycota	GS11_fam_Incertae_sedis	Endosphere	0.8165	0.0100	0.0760	No	Endosphere
Fungi	Helicoon	Ascomycota	Tubeufiaceae	Rhizosphere	0.4992	0.2470	0.3998	No	Rhizosphere
Fungi	Hirsutella	Ascomycota	Ophiocordycipitaceae	Endosphere	0.5000	0.2670	0.4005	No	Endosphere
Fungi	Hortiboletus	Basidiomycota	Boletaceae	Endosphere	0.5000	0.2580	0.3998	No	Endosphere
Fungi	Humicola	Ascomycota	Chaetomiaceae	Endosphere	0.6455	0.0600	0.2280	No	Endosphere
Fungi	Humicolopsis	Ascomycota	Helotiales_fam_Incertae_sedis	Endosphere	0.7071	0.0340	0.1436	No	Endosphere

Microbial group	Genus	Phylum	Family	Indicator group	IndVal.g	IndVal raw P	IndVal FDR	IndVal significant	Paired abundance direction
Fungi	Hymenochaetales_gen_Incertae_sedis	Basidiomycota	Hymenochaetales_fam_Incertae_sedis	Endosphere	0.7068	0.0280	0.1277	No	Endosphere
Fungi	Ilyonectria	Ascomycota	Nectriaceae	Rhizosphere	0.6054	0.5200	0.6240	No	Rhizosphere
Fungi	Inocybe	Basidiomycota	Inocybaceae	Endosphere	0.4991	0.2330	0.3998	No	Endosphere
Fungi	Keithomyces	Ascomycota	Clavicipitaceae	Endosphere	0.7638	0.0220	0.1045	No	Endosphere
Fungi	Lachnum	Ascomycota	Hyaloscyphaceae	Rhizosphere	0.4999	0.2470	0.3998	No	Rhizosphere
Fungi	Leotiomycetes_gen_Incertae_sedis	Ascomycota	Leotiomycetes_fam_Incertae_sedis	Endosphere	0.5774	0.1320	0.3344	No	Endosphere
Fungi	Leptobacillium	Ascomycota	Cordycipitaceae	Endosphere	0.7638	0.0170	0.0969	No	Endosphere
Fungi	Leptodontidium	Ascomycota	Leptodontidiaceae	Endosphere	0.5772	0.1290	0.3342	No	Endosphere
Fungi	Linnemannia	Mortierellomycota	Mortierellaceae	Endosphere	1.0000	0.0010	0.0127	Yes	Endosphere
Fungi	Meliniomyces	Ascomycota	Helotiaceae	Rhizosphere	0.5201	0.9700	1.0000	No	Endosphere
Fungi	Membranomyces	Basidiomycota	Hydnaceae	Endosphere	0.4998	0.6040	0.6955	No	Endosphere
Fungi	Metapochonia	Ascomycota	Clavicipitaceae	Endosphere	0.9574	0.0010	0.0127	Yes	Endosphere

Microbial group	Genus	Phylum	Family	Indicator group	IndVal.g	IndVal raw P	IndVal FDR	IndVal significant	Paired abundance direction
Fungi	Microbotryales_gen_Incertae_sedis	Basidiomycota	Microbotryales_fam_Incertae_sedis	Endosphere	0.7071	0.0330	0.1436	No	Endosphere
Fungi	Mortierella	Mortierellomycota	Mortierellaceae	Endosphere	0.9539	0.0020	0.0228	Yes	Endosphere
Fungi	Mortierellaceae_gen_Incertae_sedis	Mortierellomycota	Mortierellaceae	Endosphere	0.7638	0.0160	0.0969	No	Endosphere
Fungi	Mycena	Basidiomycota	Mycenaceae	Rhizosphere	0.5606	0.7780	0.8367	No	Rhizosphere
Fungi	Mycoarthris	Ascomycota	Helotiales_fam_Incertae_sedis	Endosphere	0.5000	0.2340	0.3998	No	Endosphere
Fungi	Neopestalotiopsis	Ascomycota	Pestalotiopsidaceae	Rhizosphere	0.3955	0.7710	0.8367	No	Rhizosphere
Fungi	Oidiodendron	Ascomycota	Myxotrichaceae	Endosphere	0.6789	0.5890	0.6852	No	Endosphere
Fungi	Penicillifer	Ascomycota	Nectriaceae	Endosphere	0.5000	0.2600	0.3998	No	Endosphere
Fungi	Penicillium	Ascomycota	Aspergillaceae	Endosphere	0.9997	0.0010	0.0127	Yes	Endosphere
Fungi	Pezicula	Ascomycota	Dermateaceae	Rhizosphere	0.4831	0.2560	0.3998	No	Rhizosphere
Fungi	Pezoloma	Ascomycota	Leotiaceae	Rhizosphere	0.8105	0.2080	0.3998	No	Rhizosphere
Fungi	Phialea	Ascomycota	Hyaloscyphaceae	Endosphere	0.6453	0.0630	0.2317	No	Endosphere

Microbial group	Genus	Phylum	Family	Indicator group	IndVal.g	IndVal raw P	IndVal FDR	IndVal significant	Paired abundance direction
Fungi	Phialocephala	Ascomycota	Mollisiaceae	Rhizosphere	0.9947	0.0010	0.0127	Yes	Rhizosphere
Fungi	Pleotrichocladium	Ascomycota	Melanommataceae	Rhizosphere	0.3766	0.9440	0.9964	No	Rhizosphere
Fungi	Pleuroascaceae_gen_Incertae_sedis	Ascomycota	Pleuroascaceae	Endosphere	0.5000	0.2300	0.3998	No	Endosphere
Fungi	Podila	Mortierellomycota	Mortierellaceae	Endosphere	1.0000	0.0010	0.0127	Yes	Endosphere
Fungi	Podosphaera	Ascomycota	Erysiphaceae	Endosphere	0.3659	0.5180	0.6240	No	Endosphere
Fungi	Rhizodermea	Ascomycota	Dermateaceae	Rhizosphere	0.8046	0.0200	0.1036	No	Rhizosphere
Fungi	Rozellomycota_gen_Incertae_sedis	Rozellomycota	Rozellomycota_fam_Incertae_sedis	Endosphere	1.0000	0.0010	0.0127	Yes	Endosphere
Fungi	Russula	Basidiomycota	Russulaceae	Endosphere	0.8075	0.3580	0.5123	No	Endosphere
Fungi	Sagenomella	Ascomycota	Trichocomaceae	Endosphere	0.4996	0.2470	0.3998	No	Endosphere
Fungi	Saitozyma	Basidiomycota	Trimorphomycetaceae	Endosphere	0.9868	0.0010	0.0127	Yes	Endosphere
Fungi	Scleroderma	Basidiomycota	Sclerodermataceae	Endosphere	0.5000	0.2340	0.3998	No	Endosphere
Fungi	Sebacina	Basidiomycota	Sebacinaceae	Endosphere	0.4990	0.5420	0.6436	No	Endosphere

Microbial group	Genus	Phylum	Family	Indicator group	IndVal.g	IndVal raw P	IndVal FDR	IndVal significant	Paired abundance direction
Fungi	Serendipita	Basidiomycota	Serendipitaceae	Endosphere	0.7148	0.2040	0.3998	No	Endosphere
Fungi	Serendipitaceae_gen_Incertae_sedis	Basidiomycota	Serendipitaceae	Endosphere	0.6364	0.0920	0.2913	No	Endosphere
Fungi	Solicoccozyma	Basidiomycota	Piskurozymaceae	Endosphere	0.8066	0.0140	0.0939	No	Endosphere
Fungi	Spirosphaera	Ascomycota	Tricladiaceae	Rhizosphere	0.2793	1.0000	1.0000	No	Rhizosphere
Fungi	Sporidiobolales_gen_Incertae_sedis	Basidiomycota	Sporidiobolales_fam_Incertae_sedis	Endosphere	0.6455	0.0720	0.2414	No	Endosphere
Fungi	Talaromyces	Ascomycota	Aspergillaceae	Endosphere	0.4994	0.2420	0.3998	No	Endosphere
Fungi	Teratosphaeria	Ascomycota	Teratosphaeriaceae	Rhizosphere	0.4944	0.7030	0.7935	No	Rhizosphere
Fungi	Thelephoraceae_gen_Incertae_sedis	Basidiomycota	Thelephoraceae	Endosphere	0.7635	0.0440	0.1730	No	Endosphere
Fungi	Thelonectria	Ascomycota	Nectriaceae	Endosphere	0.5000	0.2220	0.3998	No	Endosphere
Fungi	Tomentella	Basidiomycota	Thelephoraceae	Endosphere	0.4998	0.2470	0.3998	No	Endosphere
Fungi	Trechispora	Basidiomycota	Hydnodontaceae	Endosphere	0.5774	0.1190	0.3342	No	Endosphere
Fungi	Trechisporales_gen_Incertae_sedis	Basidiomycota	Trechisporales_fam_Incertae_sedis	Rhizosphere	0.5738	0.3040	0.4443	No	Rhizosphere

Microbial group	Genus	Phylum	Family	Indicator group	IndVal.g	IndVal raw P	IndVal FDR	IndVal significant	Paired abundance direction
Fungi	Trichoderma	Ascomycota	Hypocreaceae	Endosphere	0.9035	0.0010	0.0127	Yes	Endosphere
Fungi	Trogia	Basidiomycota	Tricholomataceae	Rhizosphere	0.4999	0.4880	0.6240	No	Rhizosphere
Fungi	Tuber	Ascomycota	Tuberaceae	Endosphere	0.5000	0.4990	0.6240	No	Endosphere
Fungi	Tympanidaceae_gen_Incertae_sedis	Ascomycota	Tympanidaceae	Endosphere	0.8085	0.0070	0.0665	No	Endosphere
Fungi	Umbelopsis	Mucoromycota	Umbelopsidaceae	Endosphere	0.8165	0.0080	0.0702	No	Endosphere
Fungi	unclassified_Agaricales	Basidiomycota	unclassified_Agaricales	Rhizosphere	0.2550	1.0000	1.0000	No	Rhizosphere
Fungi	unclassified_Agaricomycetes	Basidiomycota	unclassified_Agaricomycetes	Endosphere	0.6696	0.3880	0.5329	No	Endosphere
Fungi	unclassified_Apiosporaceae	Ascomycota	Apiosporaceae	Endosphere	0.5000	0.2710	0.4012	No	Endosphere
Fungi	unclassified_Ascomycota	Ascomycota	unclassified_Ascomycota	Rhizosphere	0.8648	0.2150	0.3998	No	Rhizosphere
Fungi	unclassified_Chaetomiaceae	Ascomycota	Chaetomiaceae	Endosphere	0.5774	0.1170	0.3342	No	Endosphere
Fungi	unclassified_Chaetothyriales	Ascomycota	unclassified_Chaetothyriales	Endosphere	0.6455	0.1360	0.3370	No	Endosphere
Fungi	unclassified_Didymellaceae	Ascomycota	Didymellaceae	Endosphere	0.7877	0.0770	0.2508	No	Endosphere

Microbial group	Genus	Phylum	Family	Indicator group	IndVal.g	IndVal raw P	IndVal FDR	IndVal significant	Paired abundance direction
Fungi	unclassified_Dothideomycetes	Ascomycota	unclassified_Dothideomycetes	Rhizosphere	0.7480	0.2230	0.3998	No	Rhizosphere
Fungi	unclassified_Fungi	unclassified_Fungi	unclassified_Fungi	Endosphere	0.7419	0.4960	0.6240	No	Endosphere
Fungi	unclassified_Gloniaceae	Ascomycota	Gloniaceae	Endosphere	0.4999	0.2470	0.3998	No	Endosphere
Fungi	unclassified_Helotiales	Ascomycota	unclassified_Helotiales	Endosphere	0.6714	0.3690	0.5130	No	Endosphere
Fungi	unclassified_Herpotrichiellaceae	Ascomycota	Herpotrichiellaceae	Rhizosphere	0.6932	0.7700	0.8367	No	Rhizosphere
Fungi	unclassified_Hyaloscyphaceae	Ascomycota	Hyaloscyphaceae	Rhizosphere	0.6945	0.4650	0.6164	No	Rhizosphere
Fungi	unclassified_Hypocreales	Ascomycota	unclassified_Hypocreales	Endosphere	0.7634	0.0180	0.0977	No	Endosphere
Fungi	unclassified_Leotiomycetes	Ascomycota	unclassified_Leotiomycetes	Endosphere	0.6345	0.7120	0.7958	No	Endosphere
Fungi	unclassified_Mortierellaceae	Mortierellomycota	Mortierellaceae	Endosphere	0.9107	0.0030	0.0311	Yes	Endosphere
Fungi	unclassified_Mycosphaerellales	Ascomycota	unclassified_Mycosphaerellales	Rhizosphere	0.3792	0.7470	0.8268	No	Rhizosphere
Fungi	unclassified_Ophiocordycipitaceae	Ascomycota	Ophiocordycipitaceae	Endosphere	0.4937	0.2280	0.3998	No	Endosphere
Fungi	unclassified_Orbiliaceae	Ascomycota	Orbiliaceae	Endosphere	0.3740	0.5150	0.6240	No	Endosphere

Microbial group	Genus	Phylum	Family	Indicator group	IndVal.g	IndVal raw P	IndVal FDR	IndVal significant	Paired abundance direction
Fungi	unclassified_Serendipitaceae	Basidiomycota	Serendipitaceae	Endosphere	0.7192	0.0710	0.2414	No	Endosphere
Fungi	unclassified_Sordariomycetes	Ascomycota	unclassified_Sordariomycetes	Endosphere	0.8381	0.2210	0.3998	No	Endosphere

Table S7. High-confidence compartment-associated genera supported by both IndVal and paired abundance analyses

Microbial group	Genus	Phylum	Family	Associated compartment	IndVal.g	IndVal FDR	Wilcoxon FDR	Mean rhizosphere abundance (%)	Mean endosphere abundance (%)	Mean difference (E − R, %)	Worst FDR
Bacteria	Acidothermus	Actinobacteriota	Acidothermaceae	Rhizosphere	0.9176	0.0157	0.0302	29.858	5.602	-24.256	0.0302
Bacteria	Pseudomonas	Proteobacteria	Pseudomonadaceae	Rhizosphere	0.9919	0.0190	0.0274	19.833	0.327	-19.506	0.0274
Bacteria	A21b	Proteobacteria	A21b	Endosphere	0.9574	0.0098	0.0204	0.000	0.304	0.304	0.0204
Bacteria	Acidicaldus	Proteobacteria	Acetobacteraceae	Endosphere	0.9056	0.0077	0.0242	0.020	1.277	1.256	0.0242
Bacteria	Acidipila-Silvibacterium	Acidobacteriota	Acidobacteriaceae_(Subgroup_1)	Endosphere	0.8136	0.0157	0.0358	0.047	0.355	0.308	0.0358

Microbial group	Genus	Phylum	Family	Associated compartment	IndVal.g	IndVal FDR	Wilcoxon FDR	Mean rhizosphere abundance (%)	Mean endosphere abundance (%)	Mean difference (E − R, %)	Worst FDR
Bacteria	Acidisoma	Proteobacteria	Acetobacteraceae	Endosphere	0.8840	0.0190	0.0358	0.016	0.237	0.221	0.0358
Bacteria	Actinoallomurus	Actinobacteriota	Thermomonosporaceae	Endosphere	0.8030	0.0312	0.0448	0.001	0.031	0.030	0.0448
Bacteria	Anaeromyxobacter	Myxococcota	Anaeromyxobacteraceae	Endosphere	1.0000	0.0077	0.0204	0.000	0.304	0.304	0.0204
Bacteria	B1-7BS	Proteobacteria	B1-7BS	Endosphere	0.8165	0.0232	0.0358	0.000	0.179	0.179	0.0358
Bacteria	Bauldia	Proteobacteria	Rhizobiales_Incertae_Sedis	Endosphere	0.9574	0.0098	0.0204	0.000	0.270	0.270	0.0204
Bacteria	Bdellovibrio	Bdellovibrionota	Bdellovibrionaceae	Endosphere	0.9891	0.0077	0.0204	0.005	0.236	0.231	0.0204
Bacteria	BIrii41	Myxococcota	BIrii41	Endosphere	0.8660	0.0133	0.0302	0.000	0.031	0.031	0.0302
Bacteria	Blfdi19	Myxococcota	Blfdi19	Endosphere	0.9041	0.0133	0.0242	0.003	0.134	0.131	0.0242
Bacteria	Candidatus_Berkiella	Proteobacteria	Unknown_Family	Endosphere	0.9574	0.0077	0.0204	0.000	0.069	0.069	0.0204
Bacteria	Candidatus_Ovatusbacter	Proteobacteria	Unknown_Family	Endosphere	0.8165	0.0157	0.0358	0.000	0.048	0.048	0.0358
Bacteria	Conexibacter	Actinobacteriota	Solirubrobacteraceae	Endosphere	0.9602	0.0077	0.0204	0.104	1.230	1.126	0.0204

Microbial group	Genus	Phylum	Family	Associated compartment	IndVal.g	IndVal FDR	Wilcoxon FDR	Mean rhizosphere abundance (%)	Mean endosphere abundance (%)	Mean difference (E − R, %)	Worst FDR
Bacteria	Edaphobacter	Acidobacteriota	Acidobacteriaceae_(Subgroup_1)	Endosphere	0.9129	0.0077	0.0242	0.000	0.119	0.119	0.0242
Bacteria	Ellin6067	Proteobacteria	Nitrosomonadaceae	Endosphere	0.9451	0.0098	0.0204	0.060	2.299	2.239	0.0204
Bacteria	FCPU426	FCPU426	FCPU426	Endosphere	0.7638	0.0454	0.0448	0.000	0.033	0.033	0.0454
Bacteria	Granulicella	Acidobacteriota	Acidobacteriaceae_(Subgroup_1)	Endosphere	0.9333	0.0077	0.0204	0.019	0.372	0.352	0.0204
Bacteria	Haliangium	Myxococcota	Haliangiaceae	Endosphere	0.9250	0.0133	0.0204	0.052	0.729	0.677	0.0204
Bacteria	Hirschia	Proteobacteria	Hyphomonadaceae	Endosphere	0.9129	0.0133	0.0242	0.000	0.100	0.100	0.0242
Bacteria	IMCC26256	Actinobacteriota	IMCC26256	Endosphere	0.9937	0.0077	0.0204	0.063	4.959	4.896	0.0204
Bacteria	JG36-TzT-191	Proteobacteria	JG36-TzT-191	Endosphere	0.8165	0.0174	0.0358	0.000	0.035	0.035	0.0358
Bacteria	MBNT15	MBNT15	MBNT15	Endosphere	0.8165	0.0272	0.0358	0.000	0.195	0.195	0.0358
Bacteria	MND1	Proteobacteria	Nitrosomonadaceae	Endosphere	0.9129	0.0098	0.0242	0.000	0.319	0.319	0.0242
Bacteria	MSB-4B10	Myxococcota	MSB-4B10	Endosphere	0.9129	0.0098	0.0242	0.000	0.336	0.336	0.0242

Microbial group	Genus	Phylum	Family	Associated compartment	IndVal.g	IndVal FDR	Wilcoxon FDR	Mean rhizosphere abundance (%)	Mean endosphere abundance (%)	Mean difference (E − R, %)	Worst FDR
Bacteria	Myxococcaceae	Myxococcota	Myxococcaceae	Endosphere	0.8165	0.0272	0.0358	0.000	0.083	0.083	0.0358
Bacteria	Nitrospira	Nitrospirota	Nitrospiraceae	Endosphere	0.8165	0.0232	0.0358	0.000	0.441	0.441	0.0358
Bacteria	Occallatibacter	Acidobacteriota	Acidobacteriaceae_(Subgroup_1)	Endosphere	0.9052	0.0098	0.0242	0.030	1.791	1.760	0.0242
Bacteria	Pajaroellobacter	Myxococcota	Polyangiaceae	Endosphere	0.7953	0.0436	0.0448	0.002	0.039	0.037	0.0448
Bacteria	Plot4-2H12	Proteobacteria	Sphingomonadaceae	Endosphere	0.9416	0.0098	0.0204	0.006	0.171	0.165	0.0204
Bacteria	Pseudolabrys	Proteobacteria	Xanthobacteraceae	Endosphere	0.8217	0.0174	0.0271	0.022	0.092	0.070	0.0271
Bacteria	RCP2-54	RCP2-54	RCP2-54	Endosphere	0.9908	0.0077	0.0204	0.036	1.943	1.906	0.0204
Bacteria	Reyranella	Proteobacteria	Reyranellaceae	Endosphere	0.9459	0.0098	0.0204	0.066	2.692	2.626	0.0204
Bacteria	Rhodanobacter	Proteobacteria	Rhodanobacteraceae	Endosphere	0.8627	0.0174	0.0302	0.001	0.136	0.135	0.0302
Bacteria	Rhodovastum	Proteobacteria	Acetobacteraceae	Endosphere	0.8616	0.0174	0.0448	0.013	0.103	0.090	0.0448
Bacteria	Roseiarcus	Proteobacteria	Beijerinckiaceae	Endosphere	0.9610	0.0077	0.0204	0.130	1.570	1.439	0.0204

Microbial group	Genus	Phylum	Family	Associated compartment	IndVal.g	IndVal FDR	Wilcoxon FDR	Mean rhizosphere abundance (%)	Mean endosphere abundance (%)	Mean difference (E − R, %)	Worst FDR
Bacteria	SC-I-84	Proteobacteria	SC-I-84	Endosphere	0.9574	0.0098	0.0204	0.000	0.901	0.901	0.0204
Bacteria	SM2D12	Proteobacteria	SM2D12	Endosphere	0.9887	0.0077	0.0204	0.007	0.297	0.290	0.0204
Bacteria	Subgroup_10	Acidobacteriota	Thermoanaerobaculaceae	Endosphere	0.8660	0.0190	0.0302	0.000	0.094	0.094	0.0302
Bacteria	Subgroup_15	Acidobacteriota	Subgroup_15	Endosphere	0.7638	0.0312	0.0448	0.000	0.043	0.043	0.0448
Bacteria	Subgroup_7	Acidobacteriota	Subgroup_7	Endosphere	0.7627	0.0454	0.0448	0.002	0.555	0.554	0.0454
Bacteria	SWB02	Proteobacteria	Hyphomonadaceae	Endosphere	0.8113	0.0232	0.0358	0.005	0.411	0.406	0.0358
Bacteria	TRA3-20	Proteobacteria	TRA3-20	Endosphere	0.8660	0.0174	0.0302	0.000	0.523	0.523	0.0302
Bacteria	unclassified_Acetobacteraceae	Proteobacteria	Acetobacteraceae	Endosphere	0.9853	0.0077	0.0204	0.054	1.776	1.722	0.0204
Bacteria	unclassified_Acidimicrobiia	Actinobacteriota	unclassified_Acidimicrobiia	Endosphere	0.9961	0.0077	0.0204	0.012	1.534	1.522	0.0204
Bacteria	unclassified_Acidobacteriaceae_(Subgroup_1)	Acidobacteriota	Acidobacteriaceae_(Subgroup_1)	Endosphere	0.9455	0.0077	0.0204	0.018	0.723	0.704	0.0204
Bacteria	unclassified_Acidobacteriales	Acidobacteriota	unclassified_Acidobacteriales	Endosphere	1.0000	0.0077	0.0204	0.000	0.173	0.173	0.0204

Microbial group	Genus	Phylum	Family	Associated compartment	IndVal.g	IndVal FDR	Wilcoxon FDR	Mean rhizosphere abundance (%)	Mean endosphere abundance (%)	Mean difference (E − R, %)	Worst FDR
Bacteria	unclassified_Acidobacteriota	Acidobacteriota	unclassified_Acidobacteriota	Endosphere	0.9574	0.0098	0.0204	0.000	0.066	0.066	0.0204
Bacteria	unclassified_Actinobacteriota	Actinobacteriota	unclassified_Actinobacteriota	Endosphere	0.9824	0.0077	0.0204	0.006	0.160	0.154	0.0204
Bacteria	unclassified_Alphaproteobacteria	Proteobacteria	unclassified_Alphaproteobacteria	Endosphere	0.9482	0.0077	0.0204	0.188	1.678	1.490	0.0204
Bacteria	unclassified_Bacteria	unclassified_Bacteria	unclassified_Bacteria	Endosphere	0.9762	0.0077	0.0204	0.048	0.977	0.929	0.0204
Bacteria	unclassified_Beijerinckiaceae	Proteobacteria	Beijerinckiaceae	Endosphere	0.9510	0.0077	0.0436	0.038	0.363	0.324	0.0436
Bacteria	unclassified_Burkholderiales	Proteobacteria	unclassified_Burkholderiales	Endosphere	0.9041	0.0190	0.0436	0.045	0.367	0.323	0.0436
Bacteria	unclassified_Caulobacteraceae	Proteobacteria	Caulobacteraceae	Endosphere	0.9367	0.0077	0.0218	0.153	1.094	0.941	0.0218
Bacteria	unclassified_Elsterales	Proteobacteria	unclassified_Elsterales	Endosphere	0.9574	0.0077	0.0204	0.000	0.072	0.072	0.0204
Bacteria	unclassified_Gaiellales	Actinobacteriota	unclassified_Gaiellales	Endosphere	0.9574	0.0098	0.0204	0.000	0.465	0.465	0.0204
Bacteria	unclassified_Gammaproteobacteria	Proteobacteria	unclassified_Gammaproteobacteria	Endosphere	0.8313	0.0157	0.0358	0.025	0.077	0.052	0.0358
Bacteria	unclassified_Hyphomicrobiaceae	Proteobacteria	Hyphomicrobiaceae	Endosphere	0.8165	0.0272	0.0358	0.000	0.063	0.063	0.0358

Microbial group	Genus	Phylum	Family	Associated compartment	IndVal.g	IndVal FDR	Wilcoxon FDR	Mean rhizosphere abundance (%)	Mean endosphere abundance (%)	Mean difference (E − R, %)	Worst FDR
Bacteria	unclassified_Myxococcaceae	Myxococcota	Myxococcaceae	Endosphere	0.9870	0.0077	0.0204	0.006	0.237	0.230	0.0204
Bacteria	unclassified_Rhizobiales	Proteobacteria	unclassified_Rhizobiales	Endosphere	0.9492	0.0272	0.0375	0.038	0.344	0.306	0.0375
Bacteria	unclassified_Rhodanobacteraceae	Proteobacteria	Rhodanobacteraceae	Endosphere	0.9359	0.0077	0.0204	0.005	0.113	0.108	0.0204
Bacteria	unclassified_Solirubrobacteraceae	Actinobacteriota	Solirubrobacteraceae	Endosphere	0.9388	0.0098	0.0204	0.006	0.144	0.139	0.0204
Bacteria	unclassified_Streptosporangiales	Actinobacteriota	unclassified_Streptosporangiales	Endosphere	0.8605	0.0098	0.0302	0.001	0.081	0.080	0.0302
Bacteria	unclassified_Xanthomonadaceae	Proteobacteria	Xanthomonadaceae	Endosphere	0.8165	0.0190	0.0358	0.000	0.045	0.045	0.0358
Bacteria	Variovorax	Proteobacteria	Comamonadaceae	Endosphere	0.7638	0.0449	0.0448	0.000	0.041	0.041	0.0449
Bacteria	WD260	Proteobacteria	WD260	Endosphere	0.8165	0.0217	0.0358	0.000	0.039	0.039	0.0358
Fungi	Phialocephala	Ascomycota	Mollisiaceae	Rhizosphere	0.9947	0.0127	0.0361	29.152	0.310	-28.842	0.0361
Fungi	Fungi_gen_Incertae_sedis	Fungi_phy_Incertae_sedis	Fungi_fam_Incertae_sedis	Endosphere	0.9996	0.0127	0.0361	0.002	2.284	2.282	0.0361
Fungi	Linnemannia	Mortierellomycota	Mortierellaceae	Endosphere	1.0000	0.0127	0.0361	0.000	1.773	1.773	0.0361

Microbial group	Genus	Phylum	Family	Associated compartment	IndVal.g	IndVal FDR	Wilcoxon FDR	Mean rhizosphere abundance (%)	Mean endosphere abundance (%)	Mean difference (E − R, %)	Worst FDR
Fungi	Metapochonia	Ascomycota	Clavicipitaceae	Endosphere	0.9574	0.0127	0.0422	0.000	0.607	0.607	0.0422
Fungi	Mortierella	Mortierellomycota	Mortierellaceae	Endosphere	0.9539	0.0228	0.0422	0.006	0.811	0.805	0.0422
Fungi	Penicillium	Ascomycota	Aspergillaceae	Endosphere	0.9997	0.0127	0.0361	0.004	8.630	8.625	0.0361
Fungi	Podila	Mortierellomycota	Mortierellaceae	Endosphere	1.0000	0.0127	0.0361	0.000	0.980	0.980	0.0361
Fungi	Rozellomycota_gen_Incertae_sedis	Rozellomycota	Rozellomycota_fam_Incertae_sedis	Endosphere	1.0000	0.0127	0.0361	0.000	4.352	4.352	0.0361
Fungi	Saitozyma	Basidiomycota	Trimorphomycetaceae	Endosphere	0.9868	0.0127	0.0361	0.097	3.623	3.526	0.0361
Fungi	Trichoderma	Ascomycota	Hypocreaceae	Endosphere	0.9035	0.0127	0.0481	0.023	1.105	1.082	0.0481
Fungi	unclassified_Mortierellaceae	Mortierellomycota	Mortierellaceae	Endosphere	0.9107	0.0311	0.0481	0.002	0.354	0.352	0.0481

Table S8. Final Random Forest-supported compartment-associated candidate genera with taxonomic name quality control

Microbial group	Display genus	Original genus	Phylum	Family	Associated compartment	IndVal.g	IndVal FDR	Wilcoxon FDR	Mean rhizosphere abundance (%)	Mean endosphere abundance (%)	Mean difference (E − R, %)	RF permutation importance	RF rank	Evidence tier	Name quality	Main-text candidate	Recommended location
Bacteria	unclassified_Acidimicrobiia	unclassified_Acidimicrobiia	Actinobacteriota	unclassified_Acidimicrobiia	Endosphere	0.9961	0.0077	0.0204	0.012	1.534	1.522	0.0259	1	Tier A: 3-method support	Unclassified / ambiguous	No	Supplementary - low taxonomic resolution
Bacteria	Anaeromyxobacter	Anaeromyxobacter	Myxococcota	Anaeromyxobacteraceae	Endosphere	1.0000	0.0077	0.0204	0.000	0.304	0.304	0.0242	2	Tier A: 3-method support	Standard genus name	**Yes**	Main text - preferred
Bacteria	unclassified_Bacteria	unclassified_Bacteria	unclassified_Bacteria	unclassified_Bacteria	Endosphere	0.9762	0.0077	0.0204	0.048	0.977	0.929	0.0239	3	Tier A: 3-method support	Unclassified / ambiguous	No	Supplementary - low taxonomic resolution
Bacteria	IMCC26256	IMCC26256	Actinobacteriota	IMCC26256	Endosphere	0.9937	0.0077	0.0204	0.063	4.959	4.896	0.0208	4	Tier A: 3-method support	Database / lineage placeholder	No	Supplementary - Tier A placeholder
Bacteria	unclassified_Acidobacteriales	unclassified_Acidobacteriales	Acidobacteriota	unclassified_Acidobacteriales	Endosphere	1.0000	0.0077	0.0204	0.000	0.173	0.173	0.0201	5	Tier A: 3-method support	Unclassified / ambiguous	No	Supplementary - low taxonomic

Microbial group	Display genus	Original genus	Phylum	Family	Associated compartment	IndVal.g	IndVal FDR	Wilcoxon FDR	Mean rhizosphere abundance (%)	Mean endosphere abundance (%)	Mean difference (E − R, %)	RF permutation importance	RF rank	Evidence tier	Name quality	Main-text candidate	Recommended location
																	resolution
Bacteria	Bdellovibrio	Bdellovibrio	Bdellovibrionota	Bdellovibrionaceae	Endosphere	0.9891	0.0077	0.0204	0.005	0.236	0.231	0.0191	6	Tier A: 3-method support	Standard genus name	**Yes**	Main text - preferred
Bacteria	RCP2-54	RCP2-54	RCP2-54	RCP2-54	Endosphere	0.9908	0.0077	0.0204	0.036	1.943	1.906	0.0166	7	Tier A: 3-method support	Database / lineage placeholder	No	Supplementary - Tier A placeholder
Bacteria	unclassified_Alphaproteobacteria	unclassified_Alphaproteobacteria	Proteobacteria	unclassified_Alphaproteobacteria	Endosphere	0.9482	0.0077	0.0204	0.188	1.678	1.490	0.0123	8	Tier A: 3-method support	Unclassified / ambiguous	No	Supplementary - low taxonomic resolution
Bacteria	unclassified_Acidobacteriota	unclassified_Acidobacteriota	Acidobacteriota	unclassified_Acidobacteriota	Endosphere	0.9574	0.0098	0.0204	0.000	0.066	0.066	0.0107	9	Tier A: 3-method support	Unclassified / ambiguous	No	Supplementary - low taxonomic resolution
Bacteria	Conexibacter	Conexibacter	Actinobacteriota	Solirubrobacteraceae	Endosphere	0.9602	0.0077	0.0204	0.104	1.230	1.126	0.0105	10	Tier A: 3-method support	Standard genus name	**Yes**	Main text - preferred

Microbial group	Display genus	Original genus	Phylum	Family	Associated compartment	IndVal.g	IndVal FDR	Wilcoxon FDR	Mean rhizosphere abundance (%)	Mean endosphere abundance (%)	Mean difference (E − R, %)	RF permutation importance	RF rank	Evidence tier	Name quality	Main-text candidate	Recommended location
Bacteria	SM2D12	SM2D12	Proteobacteria	SM2D12	Endosphere	0.9887	0.0077	0.0204	0.007	0.297	0.290	0.0102	11	Tier A: 3-method support	Database / lineage placeholder	No	Supplementary - Tier A placeholder
Bacteria	Bauldia	Bauldia	Proteobacteria	Rhizobiales_Incertae_Sedis	Endosphere	0.9574	0.0098	0.0204	0.000	0.270	0.270	0.0096	12	Tier A: 3-method support	Standard genus name	**Yes**	Main text - preferred
Bacteria	unclassified_Acetobacteraceae	unclassified_Acetobacteraceae	Proteobacteria	Acetobacteraceae	Endosphere	0.9853	0.0077	0.0204	0.054	1.776	1.722	0.0093	13	Tier A: 3-method support	Unclassified / ambiguous	No	Supplementary - low taxonomic resolution
Bacteria	unclassified_Actinobacteriota	unclassified_Actinobacteriota	Actinobacteriota	unclassified_Actinobacteriota	Endosphere	0.9824	0.0077	0.0204	0.006	0.160	0.154	0.0086	14	Tier A: 3-method support	Unclassified / ambiguous	No	Supplementary - low taxonomic resolution
Bacteria	A21b	A21b	Proteobacteria	A21b	Endosphere	0.9574	0.0098	0.0204	0.000	0.304	0.304	0.0079	15	Tier A: 3-method support	Database / lineage placeholder	No	Supplementary - Tier A placeholder
Bacteria	unclassified_Elsterales	unclassified_Elsterales	Proteobacteria	unclassified_Elsterales	Endosphere	0.9574	0.0077	0.0204	0.000	0.072	0.072	0.0077	16	Tier A: 3-meth	Unclassified /	No	Supplementary - low

Microbial group	Display genus	Original genus	Phylum	Family	Associated compartment	IndVal.g	IndVal FDR	Wilcoxon FDR	Mean rhizosphere abundance (%)	Mean endosphere abundance (%)	Mean difference (E − R, %)	RF permutation importance	RF rank	Evidence tier	Name quality	Main-text candidate	Recommended location
														od support	ambiguous		taxonomic resolution
Bacteria	unclassified_Acidobacteriaceae_(Subgroup_1)	unclassified_Acidobacteriaceae_(Subgroup_1)	Acidobacteriota	Acidobacteriaceae_(Subgroup_1)	Endosphere	0.9455	0.0077	0.0204	0.018	0.723	0.704	0.0074	17	Tier A: 3-method support	Unclassified / ambiguous	No	Supplementary - low taxonomic resolution
Bacteria	SC-I-84	SC-I-84	Proteobacteria	SC-I-84	Endosphere	0.9574	0.0098	0.0204	0.000	0.901	0.901	0.0073	18	Tier A: 3-method support	Database / lineage placeholder	No	Supplementary - Tier A placeholder
Bacteria	unclassified_Gaiellales	unclassified_Gaiellales	Actinobacteriota	unclassified_Gaiellales	Endosphere	0.9574	0.0098	0.0204	0.000	0.465	0.465	0.0070	19	Tier A: 3-method support	Unclassified / ambiguous	No	Supplementary - low taxonomic resolution
Bacteria	Candidatus Berkiella	Candidatus_Berkiella	Proteobacteria	Unknown_Family	Endosphere	0.9574	0.0077	0.0204	0.000	0.069	0.069	0.0060	20	Tier A: 3-method support	Candidatus genus	**Yes**	Main text - usable with Candidatus label
Fungi	Rozellomycota_gen_Incertae_sedis	Rozellomycota_gen_Incertae_sedis	Rozellomycota	Rozellomycota_fam_Incertae_sedis	Endosphere	1.0000	0.0127	0.0361	0.000	4.352	4.352	0.0438	1	Tier A: 3-method	Unclassified / ambiguous	No	Supplementary - low taxonomic

Microbial group	Display genus	Original genus	Phylum	Family	Associated compartment	IndVal.g	IndVal FDR	Wilcoxon FDR	Mean rhizosphere abundance (%)	Mean endosphere abundance (%)	Mean difference (E − R, %)	RF permutation importance	RF rank	Evidence tier	Name quality	Main-text candidate	Recommended location
														support			resolution
Fungi	Podila	Podila	Mortierellomycota	Mortierellaceae	Endosphere	1.0000	0.0127	0.0361	0.000	0.980	0.980	0.0428	2	Tier A: 3-method support	Standard genus name	**Yes**	Main text - preferred
Fungi	Linnemannia	Linnemannia	Mortierellomycota	Mortierellaceae	Endosphere	1.0000	0.0127	0.0361	0.000	1.773	1.773	0.0415	3	Tier A: 3-method support	Standard genus name	**Yes**	Main text - preferred
Fungi	Penicillium	Penicillium	Ascomycota	Aspergillaceae	Endosphere	0.9997	0.0127	0.0361	0.004	8.630	8.625	0.0379	4	Tier A: 3-method support	Standard genus name	**Yes**	Main text - preferred
Fungi	Fungi_gen_Incertae_sedis	Fungi_gen_Incertae_sedis	Fungi_phy_Incertae_sedis	Fungi_fam_Incertae_sedis	Endosphere	0.9996	0.0127	0.0361	0.002	2.284	2.282	0.0334	5	Tier A: 3-method support	Unclassified / ambiguous	No	Supplementary - low taxonomic resolution
Fungi	Metapochonia	Metapochonia	Ascomycota	Clavicipitaceae	Endosphere	0.9574	0.0127	0.0422	0.000	0.607	0.607	0.0269	6	Tier A: 3-method support	Standard genus name	**Yes**	Main text - preferred

Microbial group	Display genus	Original genus	Phylum	Family	Associated compartment	IndVal.g	IndVal FDR	Wilcoxon FDR	Mean rhizosphere abundance (%)	Mean endosphere abundance (%)	Mean difference (E − R, %)	RF permutation importance	RF rank	Evidence tier	Name quality	Main-text candidate	Recommended location
Fungi	Mortierella	Mortierella	Mortierellomycota	Mortierellaceae	Endosphere	0.9539	0.0228	0.0422	0.006	0.811	0.805	0.0233	7	Tier A: 3-method support	Standard genus name	**Yes**	Main text - preferred
Fungi	Saitozyma	Saitozyma	Basidiomycota	Trimorphomycetaceae	Endosphere	0.9868	0.0127	0.0361	0.097	3.623	3.526	0.0207	8	Tier A: 3-method support	Standard genus name	**Yes**	Main text - preferred
Fungi	Phialocephala	Phialocephala	Ascomycota	Mollisiaceae	Rhizosphere	0.9947	0.0127	0.0361	29.152	0.310	-28.842	0.0198	9	Tier A: 3-method support	Standard genus name	**Yes**	Main text - preferred
Fungi	unclassified_Mortierellaceae	unclassified_Mortierellaceae	Mortierellomycota	Mortierellaceae	Endosphere	0.9107	0.0311	0.0481	0.002	0.354	0.352	0.0105	10	Tier A: 3-method support	Unclassified / ambiguous	No	Supplementary - low taxonomic resolution
Fungi	Trichoderma	Trichoderma	Ascomycota	Hypocreaceae	Endosphere	0.9035	0.0127	0.0481	0.023	1.105	1.082	0.0062	12	Tier A: 3-method support	Standard genus name	**Yes**	Main text - preferred
